# Descriptors of Secondary Active Transporter Function and How They Relate to Partial Reactions in the Transport Cycle

**DOI:** 10.3390/membranes11030178

**Published:** 2021-03-03

**Authors:** Klaus Schicker, Shreyas Bhat, Clemens Farr, Verena Burtscher, Andreas Horner, Michael Freissmuth, Walter Sandtner

**Affiliations:** 1 Center for Physiology and Pharmacology, Division of Neurophysiology and Neuropharmacology, Medical University of Vienna, 1090 Vienna, Austria; klaus.schicker@meduniwien.ac.at; 2 Center of Physiology and Pharmacology, Institute of Pharmacology and the Gaston H. Glock Research Laboratories for Exploratory Drug Development, Medical University of Vienna, 1090 Vienna, Austria; shreyas.bhat@meduniwien.ac.at (S.B.); Clemens.Farr@meduniwien.ac.at (C.F.); verena.burtscher@meduniwien.ac.at (V.B.); michael.freissmuth@meduniwien.ac.at (M.F.); 3 Institute of Biophysics, Johannes Kepler University Linz, 4040 Linz, Austria; Andreas.Horner@jku.at

**Keywords:** secondary active transporters, solute carrier proteins, monogenetic disorders, descriptors of transport function, kinetic model

## Abstract

Plasmalemmal solute carriers (SLC*s*) gauge and control solute abundance across cellular membranes. By virtue of this action, they play an important role in numerous physiological processes. Mutations in genes encoding the SLCs alter amino acid sequence that often leads to impaired protein function and onset of monogenic disorders. To understand how these altered proteins cause disease, it is necessary to undertake relevant functional assays. These experiments reveal descriptors of SLC function such as the maximal transport velocity (V_max_), the Michaelis constant for solute uptake (K_M_), potencies for inhibition of transporter function (IC_50_/EC_50_), and many more. In several instances, the mutated versions of different SLC transporters differ from their wild-type counterparts in the value of these descriptors. While determination of these experimental parameters can provide conjecture as to how the mutation gives rise to disease, they seldom provide any definitive insights on how a variant differ from the wild-type transporter in its operation. This is because the experimental determination of association between values of the descriptors and several partial reactions a transporter undergoes is casual, but not causal, at best. In the present study, we employ kinetic models that allow us to derive explicit mathematical terms and provide experimental descriptors as a function of the rate constants used to parameterize the kinetic model of the transport cycle. We show that it is possible to utilize these mathematical expressions to deduce, from experimental outcomes, how the mutation has impinged on partial reactions in the transport cycle.

## 1. Introduction

Cellular membranes are diffusion barriers for most biologically relevant solutes. Solute carrier proteins (SLCs) are membrane proteins, which facilitate entry of many of these solutes into the interior of a cell [1,2]. While some SLCs are facilitative transporters, others harness energy contained in ionic gradients that exist across biological membranes (e.g., the gradient for Na^+^, Cl^−^, K^+^, and H^+^). The latter are referred to as secondary active transporters that establish and maintain a transmembrane gradient of the solute (i.e., substrate) they carry. Irrespective of the mechanism of transport, SLCs are essential for cell homeostasis and cell survival. They include transporters that serve as entry ports for nutrients, some that extrude toxic compounds and some that play a critical role in higher order functions such as neuronal signaling [3]. SLCs are thought to operate via the alternate access mechanism [4]. In the case of plasmalemmal SLC*s*, this mechanism posits that the substrate can either bind to the transporter at the extra- or the intracellular side, but not at both sides simultaneously. Accordingly, transport by alternate access is contingent on the ability of the solute carrier to undergo long-range conformational changes [5]; substrate (often along with co-substrate(s)) binding from the extracellular side initiates the structural rearrangements of the transporter required to transition from the outward- to the inward-facing conformation. After release of the substrate (and co-substrates) into the cytosol, the cargo-less transporter subsequently rearranges to return from the substrate-free inward to the substrate-free outward facing conformation. This sequence of events comprises the transport cycle that encompasses a closed loop of partial reactions.

Given their physiological importance, it is not surprising that many inherited monogenic diseases have been found linked to mutations in SLC-proteins. Known disorders, caused by malfunctioning carriers, range from metabolic diseases to syndromes with neurological consequences [3,6]. Associations between a genetic disease and a mutation in the allele encoding a SLC protein are largely deduced through inheritance studies of genetic linkage in families or through genome wide association studies. Molecular mechanisms that underlie function of the mutated protein and how it has digressed from that of wild-type need characterizations employing functional assays. SLC transporters that transport neurotransmitters as their solutes, for instance, are prominent examples of carriers that have been well characterized for disease-relevant variants [7]. Often, functional characterization of these transporter variants reveals one of two biological outcomes for the mutated protein: (1) some mutations lead to protein misfolding and, hence, retention of the carrier in the endoplasmic reticulum; consequently, all functions associated with this transporter are lost; and (2) other mutated transporters are properly trafficked to their target location, but fail to support normal function. These catalytic variants cause perturbations in the transport cycle of the SLCs as interpreted, in some cases, as a deviation from the values of experimentally determined ‘descriptors’ (i.e., the maximal transport velocity (V_max_), the Michaelis–Menten constant of substrate transport (K_M_), ligand inhibitory potency estimates to transporter function (IC_50_s/EC_50_s) or others, when compared to the wild-type transporter).

While changes in the values of these descriptors are attributed to occur as a result of catalytic variants, insights into how these variants differs from wild-type in kinetics of the transport cycle have remained enigmatic. In the present study, we address this question by resorting to kinetic models, which allow for predicting the outcome of experiments that functionally characterize the catalytic variants of solute carriers. We derived explicit mathematical terms, which describe the descriptors as a function of the rate constants for several partial reactions used to parameterize a kinetic model of a secondary active transporter. The outcomes from the mathematical model provide insights into the relationships that exist between the descriptors and all partial reactions a transporter can undergo. We show that it is possible to test a hypothesis, based on mathematical expression of the descriptors, which explains how mutation impinges on the kinetics of partial reactions in the transport cycle and that agrees with experimental observations.

## 2. Methods

**Figure 1:**
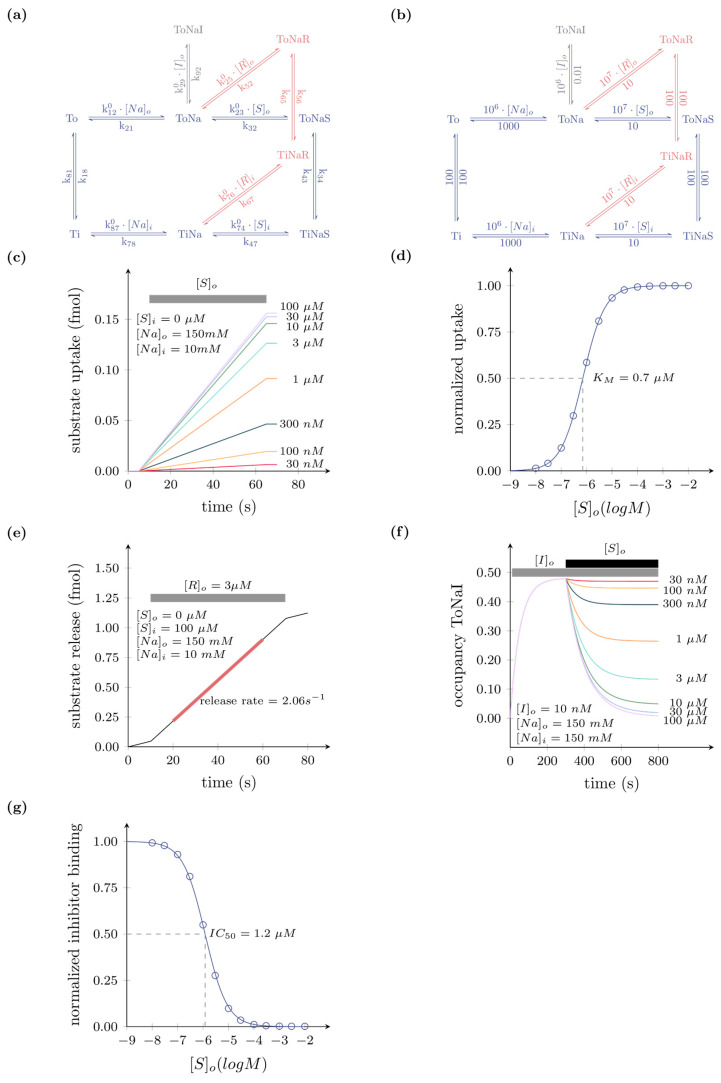
Minimal model of secondary active transporter coupled to the gradient of sodium. (**a**) Reaction schema: To, ToNa, ToNaS (in blue) are the apo, sodium, and substrate-bound outward-facing states of the transporter. Ti, TiNa, and TiNaS (in blue) are the corresponding inward states. ToNaI (in black) is the inhibitor bound state. The inhibitor binds to the outward-facing state competitively with the substrate. ToNaR and TiNaR (in red) are the releaser bound states. The releaser is an alternative substrate of the transporter. (**b**) Reaction schema in which the rate constants are parameterized with numerical values. These values were used for the subsequent simulations (**c**) Simulated substrate uptake: time-dependent substrate uptake on exposure to increasing concentrations of the substrate [S]o for 1 min. In the simulation we assumed the presence of a physiological sodium gradient and initial rate condition (i.e., [S]i = 0). (**d**) Plotted is the normalized amount of sequestered substrate after 1 min as a function of the substrate concentration. The synthetic data was fit to the Michaelis–Menten equation. The fit yielded a K_M_ of 0.708 µM. (**e**) Time-dependent substrate release on application of a releaser (3 µM). For the simulation we assumed the presence of a physiological sodium gradient. We further assumed that the cell has been preloaded with substrate upon which [S]i reached 100 µM. In the first 10 s of the simulation we did not apply the releaser. The amount of substrate released in this period reflects basal substrate release. Subsequent to this, we applied the releaser for 1 min. This accelerated the rate of substrate release. The rate of substrate release in the presence of the releaser was determined by extracting the slope of the curve from a linear fit (in red) to the data (2.06 s^−1^). (**f**) Plotted is the state occupancy of ToNaI (i.e., inhibitor bound state) as a function of time. At time point zero we applied 10 nM of the inhibitor [I]o (K_D_ = 10 nM). In the simulation we waited until the state occupancy of ToNaI had reached a steady state. Thereafter, we applied increasing concentrations of the substrate [S]o. The applied substrate competed for binding and led to a drop in the state occupancy of ToNaI. We assumed that this experiment had been conducted in a vesicular membrane preparation in which the sodium gradient had been dissipated. (**g**). Plotted in the graph is the normalized state occupancy of ToNaI as a function of the applied substrate concentration. The synthetic data was fit to the following equation: Fraction_inhibitor bound_ = 1 − ([S]_o_/IC_50_ + [S]_o_). The IC_50_ for inhibitor displacement by the substrate as determined by the fit was 1.205 µM.

Numerical simulations: Time-dependent changes in state occupancies of the model in Figure 1b were evaluated by numerical integration of the resulting system of differential equations using the Systems Biology Toolbox [8] and MATLAB 2015a (MathWorks, Natick, MA, USA).

**Figure 2:**
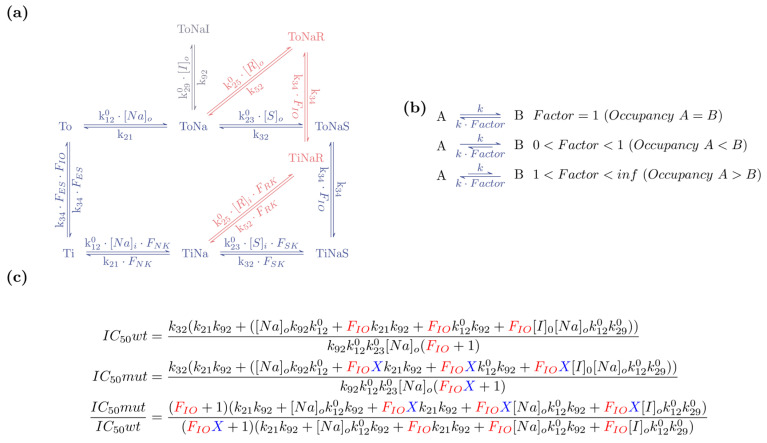
Explicit terms for descriptors of transport function. (**a**) Minimal model in a reparametrized formulation (see also Table 1). (**b**) Conformational equilibria can be gauged by the use of multiplication factors. Shown is a two state model. If the *Factor* is 1 the state occupancies of A and B are equal (upper panel). If the *Factor* is smaller than 1 the state occupancy of A is smaller than the one of B (middle panel). At values larger than 1 the situation is opposite (i.e., A > B; lower panel). (**c**) The upper panel shows the term which gives the IC_50_ for inhibitor displacement by the substrate as a function of the rate constants used to parameterize the model in (**a**). This term applies to wild-type (IC_50_wt). In the term describing the IC_50_ for the mutant (IC_50_mut), we introduced an additional factor X, which we multiplied with the factor F_IO_ (middle panel). In the lower panel we show the ratio of the two terms (i.e., IC_50_ mut/IC_50_ wt).

**Computer assisted algebra:** To evaluate the expressions in Figure 2c, we used the *isAlways* function contained in the symbolic toolbox of Matlab and the *TrueQ* function in combination with the *Refine* function in Mathematica.

Nonlinear constrained multivariate optimization algorithm: To minimize or maximize algebraic equations, we employed the *fmincon*- solver contained in Matlab. We used linear and non-linear constraints to restrict the rate constants of the model in Figure 2a to realistic values. We constrained the rates constants as follows: (i) the association rate constants for Na^+^, substrate and releaser were allowed to adopt values between 10^3^ M^−1^∙s^−1^ and 10^8^ M^−1^∙s^−1^ (i.e., diffusion limit); (ii) the corresponding dissociation rate constants were allowed to adopt values between 0.1 s^−1^ and 10^5^ s^−1^; (iii) we also constrained the affinities of Na^+^, substrate and releaser: 100 µM < [Na^+^] < 100 mM; 10 nM < [S] < 10 mM; 10 nM < [R] < 10 mM; and (iv) the rate constants for the conformational transitions were constrained to values between 0.1 s^−1^ and 10^5^ s^−1^.

## 3. Results

### 3.1. The Minimal Model of Secondary Active Transporter

In Figure 1a, we show the kinetic scheme of the minimal model of a secondary active transporter. We assumed that the hypothetical transporter is energetically driven by the transmembrane gradient of sodium (Na). Accordingly, the scheme includes the apo-outward-facing state (To), the sodium-bound outward-facing state (ToNa), the ternary complex of the outward-facing state with sodium and substrate (ToNaS), and, in addition, the corresponding inward-facing states (Ti, TiNa, and TiNaS). The reactions connecting these states form a closed loop, which is referred to as the transport cycle. A kinetic model containing only these six states (shown in blue) is sufficient to predict substrate uptake through a transporter. However, substrate uptake is not the only aspect of transporter function, which is typically assessed when characterizing a secondary active transporter or mutated versions thereof. Other assays test the ability of a transporter to bind an inhibitor (e.g., K_D_ of an inhibitor, measured in binding experiments) or measure release of cytosolic substrate on application of a releaser (i.e., amount of cytosolic substrate released on application of a synthetic or endogenous substrate). To account for these functions, we included an inhibitor bound state (ToNaI) and releaser bound states (ToNaR and TiNaR). The resulting addition of these states to the ones, which account for substrate uptake, allow for modelling the outcome of a broad range of transporter assays. We stress that this scheme is a minimal model and simplistic. In reality, secondary active transporters frequently function in ways that are more complex and require a comprehensive reaction schemes for faithful representation of transporter function. For instance, some utilize additional co-substrates such as chloride or potassium [9,10], while some may bind substrates and co-substrates in a random rather than a sequential order [11,12] or some may display ionic slippage [13,14]. In addition, many transporters are voltage-dependent. This voltage dependence can be the result of the binding of charged (co)substrate within the membrane electric field or conformational rearrangements, which move charge(s) through the electric field. However, the partial reactions associated with charge movement can differ between different transporters [12]. Hence, we refrained from incorporating arbitrary assignment of voltage dependence into the minimal model.

### 3.2. Kinetic Models can Predict Experimental Outcomes

We parameterized the model in Figure 1a with a plausible set of rate constants (see Figure 1b). We assumed that ligands bind to the hypothetical transporter with rates that are not limited by diffusion [15,16]. As shown in Figure 1c–g, this minimal model is sufficient to predict the outcome of a wide range of experiments, which are typically performed to explore the function of a solute carrier. For instance, in Figure 1c we show the result of an in silico experiment in which we simulated the time-dependent substrate uptake on application of increasing substrate concentrations. We note that for the simulation, we assumed the intracellular substrate concentration (S_in_) as zero (i.e., initial rate condition) and that the substrate was applied extracellularly for 1 min. Substrate uptake was modelled as the substrate dissociation from the inward-facing state: (k_47_*TiNaS). In Figure 1d, we plotted the amount of substrate that accumulated in the cell within 1 min as a function of the applied substrate concentration. We extracted the K_M_ for substrate uptake as ~700 nM by fitting the synthetic data to the Michaelis–Menten equation. In Figure 1e, we conducted an in silico substrate release experiment. For the simulation, we further assumed that the cell had prior been loaded with substrate, upon which the intracellular substrate concentration reached 0.1 mM. The releaser, assumed to be an alternative substrate, can readily exchange with the substrate, which was accumulated within the cell, and thus lead to its release. Substrate release was modeled as the dissociation of the substrate from the outward-facing state (k_32_×ToNaS). As shown in Figure 1e, substrate is released over time upon extracellular application of a releaser (3 μM). From the slope of the curve, we determined the rate of substrate release as ~2 s^−1^. The illustration in Figure 1e shows that substrate is initially released at a lower basal rate prior to the application of the releaser. In Figure 1f, our kinetic model also emulated a radioligand binding experiment. The radioligand was assumed to be a transport incompetent inhibitor. We further assumed that the experiment was conducted in a vesicular membrane preparation in which there was no sodium gradient. In the simulation, we set the K_D_ of the inhibitor to 10 nM. Figure 1f shows the state occupancy of ToNaI over time. In the in silico experiment, we waited until inhibitor binding had reached steady state (i.e., time-independent state occupancy), before we applied increasing concentrations of the substrate that competed for binding to the same transporter. The substrate binds to and prevents the inhibitor from forming complexes with the transporter, which results in a decrease in the state occupancy of ToNaI. At higher concentrations of the substrate, inhibitor binding to the transporter was reduced. In Figure 1g, we plotted the state occupancy of ToNaI as function of the applied substrate concentration ([S]_o_ ranging from 10 nM to 100 μM). We extracted the potency for inhibitor displacement by the substrate (IC_50_ = ~1 μM), by fitting the synthetic data to the appropriate function (see legend in Figure 1).

### 3.3. Explicit Mathematical Expressions Defining Descriptors

The synthetic data in Figure 1 was obtained by numerically solving the system of differential equations, which constituted the kinetic model. We show that descriptors (e.g., K_M_, rate of release, IC_50_, etc.) determined in experiments can be extracted from the model by emulating the actual experiments. However, when using this approach, it remains unclear how the descriptors depend on specific partial reactions, which a transporter can undergo, when performing a particular transporter function. In other words, it is not readily evident how a change in a rate constant parameterizing the model will impinge on the value obtained for a given descriptor. This information, while contained in the model, remains implicit. As a remedy to this problem, we searched for ways to derive explicit terms, which provided the descriptors as a function of the rate constants. Most descriptors determined in experiments on solute carriers fortunately report on functions of the transporter at steady state (i.e., when state occupancies have reached time independence). Thereby, they fulfill an important criterion to be derived by an approach first described by King and Altman [17] and later extended by Hill [18]. This method makes it possible to obtain analytical expressions for the state occupancies at steady state as well as net- fluxes through loops in kinetic models (see Appendix A). We used this method to derive the defining terms for the K_M_ and V_max_ of substrate uptake_,_ the rate of substrate release and IC_50_ for inhibitor displacement by a substrate (see Appendix A). The equations of these terms are provided for in the Supplementary Data. The validity of these derived expressions was verified by plugging in numeric values for the rate constants (i.e., those used in Figure 1b) and by comparing the calculated values for the descriptors with those obtained from the corresponding simulations. We found the calculated value and the value obtained from the simulation were in excellent agreement (i.e., the values differed from each other only starting at the 4th digit after the decimal. This was presumably due to rounding errors).

### 3.4. Experimental Outcomes of Descriptors Offer Diagnosis to the Type of Functional Defect Caused by a Mutation

When subjected to experimental scrutiny, mutated transporters typically deviate from their wild-type counterparts in the value of their descriptors. Herein, we describe an approach that utilizes the explicit mathematical expressions for the descriptors to deduce how a mutation impinges on partial reactions in the transport cycle. For this purpose, we first redefined the rate constants in the scheme of Figure 1a as shown in Table 1. Figure 2a shows the reparametrized version of the scheme in Figure 1a. The introduced multiplication factors (e.g., F_IO_, F_ES_, etc.) can adopt values between 0 and infinity. In this formulation, microscopic reversibility is always maintained on parametrization of the variables with numeric values (i.e., the product of the rate constants in the forward direction of a loop match the product of the rate constants in the opposite direction). Importantly, this formulation does not exclude valid sets of rate constants; as a result this model remains applicable to every transporter that operates according to the scheme, i.e., the minimal model. However, the formulation does not allow for parameter sets in which the extra- and intracellular affinity for sodium and substrate differ from each other, respectively. This restriction can be overcome by introducing additional multiplication factors, which we omitted for the sake of simplicity. As shown for a simple two state model (Figure 2b), changes in the multiplication factors alter state occupancies at steady state, because these depend on the ratios of the rates of transitions entering and leaving the state. Thus, if in the model in Figure 2a F_IO_ assumes the value of 1, the outward- and the inward-facing states are equally stable. If, however, F_IO_ assumes values between 0 and 1 the conformational equilibrium of the transporter is biased towards the inward-facing state. In contrast, at values larger than 1 the transporter is stabilized in the outward facing conformation. Similarly, when F_ES_ assumes a value between 0 and 1, the substrate-free states are stabilized. Conversely, at values larger than 1, the substrate-bound states are more frequently occupied. The factors F_NK_, F_SK_ and F_RK_ influence the kinetics of binding reactions and can be interpreted as defining the accessibility to the corresponding binding sites. For instance, if F_SK_ adopts values between 0 and 1, the binding site for the substrate on the intracellular side is less accessible than the binding site at the extracellular side. At values larger than 1 the situation is opposite. In this framework, changes in transporter function, which are caused by a mutation, can be thought to occur because one or more of these multiplication factors has adopted a different value than in the wild-type transporter.

We next show that it is possible to mathematically phrase questions as to how the mechanics of the mutant may have changed when considering experimental outcomes with the formulation chosen in Figure 2a.

Let us consider an example in which we experimentally find the IC_50_ for the displacement of an inhibitor by a substrate is larger for the mutant than for wild-type. Let us further assume that we know that the inhibitor binds to the orthosteric binding site in the outward facing conformation. We can then ask, if this mutation shifts the equilibrium towards the inward-facing state. In Figure 2c we show the term, which gives the IC_50_ for inhibitor displacement by the substrate as a function of the rate constants, multiplication factors and ligand concentrations used in the reparametrized formulation of Figure 2a. The term in the upper panel applies to wild-type (i.e., IC_50_wt). In the term, which applied to the mutant (i.e., IC_50_mut), we introduced an additional factor (X), which we multiplied with F_IO_ (middle panel). It is evident that IC_50_wt and IC_50_mut are identical, if X assumes a value of 1. If, however, X is smaller than 1 the product of F_IO_ and X will be smaller than F_IO_ alone. Hence, in this situation the mutated transporter is more biased to the inward facing conformation than its wild-type counterpart. In the lower panel of Figure 2c we show the ratio of the two terms (i.e., IC_50_mut/IC_50_wt).

By utilizing this ratio we can now phrase the above question mathematically: we can put forth the proposition that the ratio can be larger than 1 (i.e., the mutant has a higher IC_50_ than the wild-type) for X lower than 1 (i.e., the mutant is more biased to the inward facing conformation than wild-type transporter). It is already difficult to accept or refute this proposition for the comparably simple equation shown in Figure 2c. However, by applying computer assisted algebra, it is possible to prove that this statement is false: there is no solution within the parameter space of the model, which allows for the ratio to be larger than 1 when X is smaller than 1. We can thus conclude that a mutant with these properties does not shift the equilibrium to the inward-facing state.

Admittedly, this is a trivial example. It is easy to understand that a mutation, which leads to the stabilization of the inward facing conformation, is unlikely to decrease the ability of a substrate to displace an inhibitor, which shows a preference for the outward open conformation. However, the same approach can be used to address more complex questions.

For example, let us consider a mutation, which enhances substrate release: i.e., upon application of a releaser, substrate efflux by the mutated transporter is larger than that mediated by the wild-type transporter. We can again ask, if this mutant has shifted the equilibrium to the inward facing conformation. For this, we can state a ratio analogous to the one shown in Figure 2c by using the analytical expression for the rate of substrate release. We can mathematically phrase this question as follows: is the ratio larger than 1 (i.e., mutant releases more substrate than wild-type), if X is smaller than 1 (i.e., the mutated transporter is more biased to inward facing conformation than wild-type). Unfortunately, neither of the two tested computer algebra systems (Matlab and Mathematica) were able to decide, whether this statement is true or false. Presumably, this was because the mathematical expression for the ratio is too large and complex (see supplement). For this reason, we resorted to an alternative, approach, in which we employed a nonlinear constrained multivariate optimization algorithm (e.g., the *fmincon* function in Matlab). *fmincon* searches for the minima of a function and allows for imposing constraints on variables subjected to optimization. It is also possible to use *fmincon* to find the maxima of a function by minimizing its inverse function. In the specific example considered here, it is the ratio (rate of release mut/rate of release wt) which we want to minimize or maximize. This ratio is a function of the following list of model parameters: F_ES_, F_IO_, F_NK_, F_RK_, F_SK_, X, k^0^_12_, k_21_, k^0^_23_, k^0^_25_, k_32_, k_34_, k_52_, [Na]_o_, [Na]_i_, [R]_o_, [S]_I_, which are the variables subjected to optimization. As the derived term for the ratio does not harbor critical points (i.e., the gradient of this multivariable function, is at no point, zero (data not shown)) the optimizer returned a different parameter set in every run (i.e., only finding local minima/maxima). This, however, is good enough for our purpose, because finding one valid example (parameter set) is sufficient to prove the tested hypothesis.

To answer the above question we searched for the maxima of the ratio (i.e., the release is larger in the mutated transporter), while constraining X to only adopt values smaller than 1 (i.e., the mutation has shifted the equilibrium to the inward-facing state). We further ensured that the optimizer only returned realistic parameter sets by imposing additional constraints (see methods). These prevented, for example, the on-rates for sodium, substrate, and the releaser exceeded the diffusion limit. The optimizer consistently returned parameter sets, which resulted in the ratio being larger than 1. It is thus conceivable that the mutated transporter is more inwardly biased than the wild-type transporter.

We can also test whether our experimental observation is commensurate with the idea that the mutated transporter is more biased to the outward facing conformation. For this we can constrain X to values larger than 1 (i.e., the mutant is more biased to the outward facing conformation in comparison to wild-type). Additionally, under these conditions the optimizer returned values for the ratio, which were larger than 1. Hence, the available information is insufficient to decide, whether the mutant has shifted the equilibrium to either one of the two conformations.

### 3.5. Anomalous Substrate Release

The monoamine transporters are the target of amphetamines. Amphetamines are examples of previously mentioned ‘releasers’; they elevate the extracellular monoamine concentration of dopamine, norepinephrine, and serotonin, respectively, by facilitating release of intracellular monoamines via the corresponding transporters by an exchange mechanism. In this context, it is worth mentioning that amphetamine induced substrate release was previously proposed to also occur via a transporter channel mode [19]. However, as most experimental observations regarding release can be parsimoniously explained by simple exchange [11,20], we refrained from considering other complex models. Several studies have described mutated versions of the dopamine transporter (DAT), which showed a property termed “anomalous release” [21,22,23]. These mutants showed less substrate release in the presence of amphetamine than it is absence. This is opposite to how amphetamines act on wild-type DAT.

**Figure 3:**
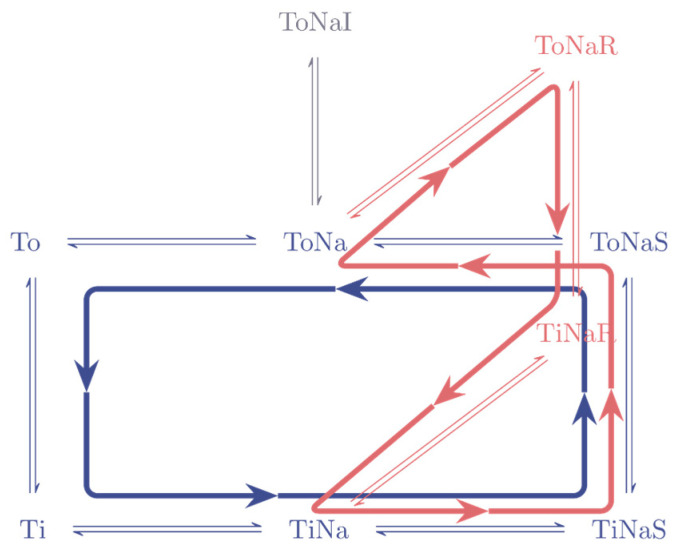
Anomalous release. The two paths in the reaction diagram, which give rise to substrate release. The cycle in blue is responsible for basal substrate release (substrate release in the absence of a releaser). The cycle in red indicates the path via which the substrate leaves the cell interior when a releaser is present.

Both phenomena, amphetamine-induced release and anomalous release, can be readily understood on inspection of the minimal model of a secondary active transporter. The reaction scheme details two alternative paths through which a substrate molecule can be released extracellularly from the cell interior. One path is described by the reactions, which point into the counter clockwise direction of the transport cycle (indicated by the blue arrow in Figure 3). This path is responsible for “basal substrate release“ (i.e., substrate release, which occurs in the absence of amphetamine). The second path (indicated by the red arrow) gives rise to substrate efflux upon application of a releaser. It is described by a sequence of reactions as follows. The amphetamine binds to the outward facing conformation upon which it is carried through the membrane. It then dissociates into the cytosol, which allows for binding of intracellular substrate. In a subsequent step, dopamine is transported to the extracellular side, where it can dissociate from the transporter. In wild-type DAT, the net substrate flux through the second path is larger than basal substrate release. As a consequence, more dopamine is released in the presence of amphetamine than in its absence. In the mutants, however, the situation is opposite. Here, the basal substrate efflux is larger than that through the second path. For this reason, the application of an amphetamine decreases the amount of released substrate.

As mentioned earlier, using the King–Altman method and its extensions by Hill, it is possible to obtain mathematical expressions that give the net flux through a kinetic cycle as a function of the defining rate constants. We used this method to derive the equations for the net fluxes through the paths responsible for basal substrate release and amphetamine induced substrate release, respectively. Owing to the complexity of the equations, we show them in the Supplementary Data and abbreviate them here as A (i.e., the rate of basal substrate release) and B (i.e., the rate of substrate release induced by a releaser). We then used these mathematical expressions to ask the following question: Is it possible that anomalous release is the consequence of the mutation having biased the transporter to the inward facing conformation? We mathematically phrased this question by again introducing a factor X (see above), which we multiplied with the factor F_IO_ in the terms of A and B, which apply to the mutated transporter (A_mut_ and B_mut_). We then asked if (B_mut_-A_mut_)*(1-A_wt_/B_wt_) can be smaller than zero, provided that X is smaller than 1 (i.e., the mutated transporter is more biased to inward facing conformation than wild-type). The above expression assumes values smaller than zero, if the following condition is met: in the wild-type transporter application of a releaser leads to increased substrate release, but to a decreased substrate release in the mutant (i.e., anomalous substrate release). We used the *fmincon* function to find parameter sets, which fulfill this condition. However, even after running *fmincon* 100,000 times, it never returned values smaller than zero for the above term. We therefore consider it unlikely that mutations, which shift the conformational equilibrium towards the inward-facing state can support anomalous substrate release. However, we concede the flaw in this argument: it is possible that such parameters sets exist but that *fmincon* was unable to find them. We note that, in the absence of any constraint other than the constraint on X, the optimization function can find combinations of rate constants for which the above term assumed values smaller than zero. However, in these instances, the resulting rate constants were never reasonable; the on-rates values adopted exceeded the diffusion limit and/or the affinities for the substrates and sodium were unrealistically high or low. In contrast, when we asked whether an outwardly biased mutant could support anomalous release, *fmincon* returned reasonable parameter sets that met this assumption in every run. We thus believe that a mutant, which is biased to the outward facing conformation, is more likely to support anomalous substrate release.

## 4. Discussion

Kinetic models can predict the outcome of experiments, which are typically performed to characterize the function of a solute carrier. However, the relations between the descriptors of transporter function and the partial reactions, which a transporter can undergo, remain implicit if the system of differential equations that constitute the kinetic model is solved numerically. As a remedy to this problem, we employed a method described by King and Altmann [17], which allows for deriving explicit mathematical definitions of the descriptors as a function of the rate constants used to parameterize the model. We limited our analysis to the minimal model of a secondary active transporter. We emphasize, however, that the corresponding terms can also be derived for larger reaction schemes. The mathematical expressions that apply to the minimal model are already exceedingly complex and they are more so for more realistic models. Their complexity precludes their intuitive understanding. It is nevertheless possible to use them to gain insights into the logic of substrate transport through secondary active transporters. In the present study, we show that explicit mathematical terms can be employed to examine mechanistic interpretations for the effect of mutations: the hypothetical explanation must show how a given mutation impinges on partial reactions in the transport cycle and it must be consistent with the available experimental information. We demonstrated this with three different functional aspects of a secondary active transporter. This methodology, however, can also be further expanded to ask questions that are far more sophisticated. For example, mathematical terms of different descriptors can be subjected to conjunction. This conjunction defines a mathematical proposition and provides the mathematical framework to address the question, how a set of observations agrees with a given hypothesis. Moreover, it can also be envisaged that the effect of the mutation on the transport cycle is more complex (i.e., the resulting functional change is not accounted for by adapting only one of the multiplication factors specified in the re-parameterized formulation). This can be addressed by introducing additional multiplication factors (e.g., ‘Y’ and ‘Z‘ akin to previously described ‘X’). These account for mutation-induced shifts in the abundance of conformational species (e.g., F_ES_) and changes in other descriptors (e.g., F_SK_ etc.). It is conceivable that mutations alter the voltage dependence of the transport cycle that may, in turn, change the values of descriptors. Although voltage dependence was not incorporated in our minimal model, we emphasize that this can be easily accounted for (see Appendix A). The effect of voltage on the function of a transporter depends on which partial reaction(s) in the transport cycle carry charge. Since this can be different for different transporters, generic assessment of this variable is difficult. Yet, if the voltage-dependent reactions for the transporter under scrutiny are known, our minimal model can test, if the mutation impinged on these reactions in a manner commensurable with available experimental data.

The approach has the shortcoming that the explicit expressions for the descriptors, which were derived and tested in this study, only apply to transporters operating according to the scheme shown in Figure 1a (i.e., the minimal model). Conclusions drawn from this model may not apply to a transporter, which requires a more complex scheme for a faithful description of its function. Unfortunately, we could not come up with a method to judge the generality of a statement, which for the minimal model, we found true or false. However, when we derived the explicit expression for the corresponding observables for a kinetic model published for DAT [24], we obtained the same answers to the three questions explored above, i.e., (i) ratio of IC_50_-mutant/IC_50_wt being incompatible with an inward facing bias; (ii) higher release of the mutant being compatible with both an inward and outward facing bias; and (iii) anomalous release being exclusively compatible with an outward facing bias of the mutant (data not shown). The published model for DAT only differs from the minimal model in that it also accounts for chloride binding. We therefore suspect that conclusions drawn from the minimal model also hold true for models, which are more complex. In this context, it is worth mentioning that one of the three disease relevant mutations in DAT [21,22,23], which have anomalous substrate release (i.e., DAT-T356M) was shown to be biased to the outward-facing state by two different experimental approaches [25,26], as predicted by our model. The other two DAT mutants (A559V and D421M) were not explored for their conformational bias. Based on our model, we predict that they have the same conformational bias as DAT-T356M: the A559V and D421M variants are also predominantly trapped in the outward-facing state and less likely to visit the inward facing conformation.

An implicit assumption in our approach is that mutant and wild-type adhere to the same reaction scheme. In this view, mutant and wild-type differ from each other in the specific values of the rate constants used to parameterize the kinetic model. One could argue, however, that a change caused by a mutation could be more severe. For instance, a mutated transporter might feature enhanced sodium slippage (i.e., the mutant can move from the inward to the outward facing conformation in the presence of sodium but in the absence of substrate) than seen in its wild-type counterpart [14]. The minimal model cannot account for this property and would necessitate different reaction schemes for mutant variants and wild-type transporter. However, the minimal model can simply be expanded by incorporating the reaction needed to model sodium slippage (i.e., a reaction connecting ToNa and TiNa in the minimal model). If there is no experimental evidence for sodium slippage in the wild-type transporter, the rate constants, which parameterize this reaction, can be constrained to values close to zero for the descriptors of the wild-type transporter. Hence, it is possible to generalize the reaction scheme such that it applies to both mutant and wild-type transporters. In this way, the method remains applicable even for mutants with novel properties, which are not observed in the wild-type transporter. The underlying assumption is that the gain-of-function in the mutant amplifies a partial reaction, which is also present in the wild-type transporter albeit at a very low probability. In this context, it is worth pointing out that the fidelity of this method can be improved by having a more detailed knowledge on all partial reactions occurring in the catalytic cycle of the wild-type transporter. In the examples mentioned in the result section, we imposed relatively mild constraints on the values a rate constant can adopt (see method section). These constraints can be narrowed down and their accuracy improved based on kinetic information obtained from experiments with better temporal resolution (e.g., electrophysiological recordings) conducted on wild-type transporters [12,24,27,28]. The resulting additional information may lead to rejecting a hypothesis, which—in the absence of realistic kinetic data—would be considered viable.

Disease relevant mutations in SLC transporters cause non-synonymous changes in the amino acid sequence. Some changes lead to misfolding of the protein and cause retention of the transporter in the endoplasmic reticulum, resulting into a loss of function phenotype [29]. In contrast, mutated transporters, which are delivered to their target location (e.g., plasma membrane), can feature a more intricate pattern of change [7]. The possibilities range from loss- to gain-of-function but also to a loss in one but gain in another function (e.g., an increase in basal substrate release but decrease in substrate uptake). One way to gather information on the functional change caused by the mutation is to compare parameters obtained from experiments conducted on both mutant and wild-type transporters. The outcome of these experiments can inform hypotheses on the mechanism, which result in the phenotypic change causing the disease. However, they provide limited insights into the mutation-induced change in the transport cycle. This understanding is important to pave a way to a therapy. This is highlighted by the observations that the transport defect in the mutant DAT-T356M can be corrected by zinc [26], which acts as an allosteric modulator of the transport cycle [30]. Allosteric ligands can be discovered by relying on serendipitous discovery using high-throughput screens [31]. It is, however, more gratifying to approach the problem by rational drug design based on an in-depth understanding of the aberrant transport cycle.

The method described in the present study, capitalizes on the availability of explicit expressions for parameters determined in experiments. The currently described methods based on venerable approach described by King and Altmann [17] are, to the best of our knowledge, the firsts to derive and obtain these explicit terms. Encoded in the terms for the descriptors are the rules, which govern the function of solute carriers. However, due to their complexity, these rules are difficult to decipher. We are confident that deeper insights can be obtained in the near future given the computational power of today’s computers and the ease by which sophisticated mathematical operations/concepts can be implemented with the help of suitable software packages. It is currently not clear, whether an approach exists, which allows for deciding if and when a statement proven for one model can be extrapolated to a larger set of models. This is an example of a question worth addressing.

## 5. Conclusions

We show a method to derive mathematical terms, which describe typically employed descriptors of transport function (e.g., K_M_, V_max_ of substrate uptake, etc.) and how they relate to the partial reactions in the transport cycle. This method can be applied in two ways: (i) as a prognostic tool to predict how altered partial reactions affect the values of these descriptors; and (ii) as an analytic tool to judge from experimental observations, which partial reaction(s) is most likely altered in a given catalytic variant of a transporter.

## Figures and Tables

**Table 1:** Reparameterized rates.

Original	Reparameterized
k_43_	k_34_ × F_IO_
k_18_	k_34_ × F_ES_
k_81_	k_34_ × F_IO_ × F_ES_
k_56_	k_34_
k_65_	k_34_ × F_IO_
k^0^_76_	k^0^_25_ × F_RK_
k_52_	k_34_ × F_RK_
k^0^_87_	k^0^_12_ × F_NK_
k_78_	K_21_ × F_NK_
k^0^_74_	k^0^_23_ × F_SK_
k_47_	k_32_ × F_SK_

We obtained the reparameterized formulation shown in Figure 2a by replacing the rate constants in Figure 1a as shown in the table.

**Figure A1:**
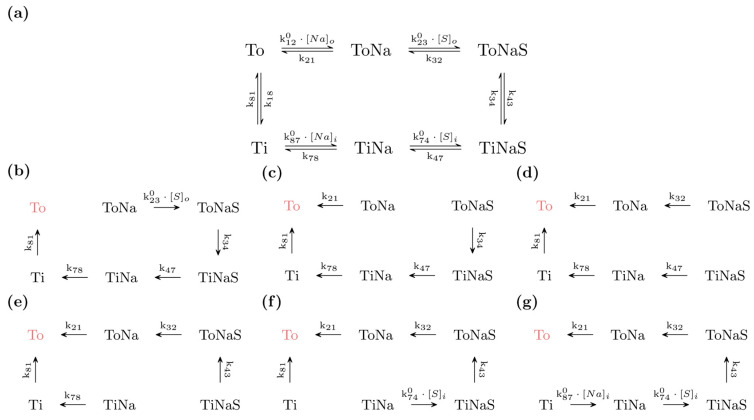
King and Altmann approach. (**a**) Reaction schema of the substrate uptake cycle of the minimal model of a secondary active transporters. (**b**–**g**) shows all directional diagrams (paths) to state To (in red).

## Supplementary Materials

The following are available online at https://www.mdpi.com/2077-0375/11/3/178/s1, the supplement contains the explicit terms for V_max_, K_M_, IC_50_ for displacement of an inhibitor by substrate, and the rate of substrate release in the original formulation and for the ratio Release_Mut_/Release_wt_ as well as for the term: B_mut_-A_mut_*(1-A_wt_/B_wt_) in the reparameterized formulation.

## Appendix A. Derivation of the Explicit Terms for State Occupancies and Net-Fluxes though Cycles at Steady State

The “diagram method” allows for calculating steady state occupancies of states specified in a reaction diagram. The principle of this method was first described in 1956 [17] and has since been refined and extended by Terrell Hill [18]. Here, we present the method in its fundamental form. For further information, please refer to [18].

In Figure A1a, we show the loop of the model that gives rise to substrate uptake. To calculate the steady state occupancy of a state in this cycle, it is necessary to find all paths in the scheme, which lead to this state, and that are one reaction short of closing the loop (i.e., directional diagrams). Once these paths have been identified, we must form the product of the involved rate constants for each individual path. This is repeated for all states in the scheme. The steady state occupancy of a state is then given by the sum of all paths (each path is the product of rate constants associated with accompanying states) leading to this state divided by the sum of all possible paths as determined by the product of the rates involved in all directional diagrams for each state. An example is shown for the state To in Figure A1b–g. Each figure shows a directional diagram of a path leading to To. The product of the rates of the path specified in Figure A1b is: k^0^_23_ × [S]_o_ × k_34_ × k_47_ × k_78_ × k_81_. For the path shown in Figure A1c it is k_21_ × k_34_ × k_47_ × k_78_ × k_81_ and so forth.

(A1) $$p_{To}=\frac{sum\;of\;directional\;diagrams\;leading\;to\;state\;To}{sum\;of\;all\;possible\;directional\;diagrams}$$

Since the number of directional diagrams surges with the complexity of the model, their compilation is tedious and error prone. Therefore, we used an algorithm published by Cornish-Bowden, which allows for automating the search for the directional diagrams [32]. We implemented this algorithm in Matlab (the Matlab scripts are available upon request)

The explicit terms for the state occupancies are not sufficient to derive mathematical expressions of many descriptors of transport function (e.g., K_M_, V_max_ and the rate of substrate release). For these, we need to obtain expressions describing net-fluxes through loops in the reaction diagram. These can be obtained as follows:

For the loop depicted in Figure A1a, the netflux (J) is given by the product of the rates in the clockwise minus the product of the rates in the counter-clockwise direction of the cycle divided by the sum of all possible directional diagrams (i.e., the sum of product of the rates involved in all directional diagrams for each state):

(A2) $$J\;=\;\frac{\prod_{l}^{+}-\prod_{l}^{-}\;}{\sum }$$

In more complicated cases, like for the scheme depicted in Figure 1a in the main text, one has to include additional paths that have been incorporated into the transport cycle (e.g., ToNaI -> ToNa leads into the substrate uptake cycle). We must account for this by multiplying the netflux by the sum of all possible directional diagrams leading into the cycle:

(A3) $$J\;=\;\frac{\left(\prod_{l}^{+}-\prod_{l}^{-}\right)\sum_{l}^{}}{\sum }$$

**Derivation of the explicit terms for descriptors of transporter function**

V_max_ is the maximum rate of substrate uptake a transporter can support. The uptake rate is maximal when binding of extracellular substrate is not rate limiting. This is the case when the extracellular substrate concentration [S]o is high. Accordingly, V_max_ is the limit of the net uptake flux equation for [S]o approaching infinity. We assumed that, in uptake experiments, the intracellular substrate concentration [S]i) is zero. We therefore substituted in the resulting terms [S]i with zero. The explicit term for V_max_ can be found in the supplement.

We obtained the term for K_M_ by solving for the substrate concentration [S]o at which the uptake rate is V_max_/2. The explicit term for K_M_ can be found in the supplement.

To describe inhibitor binding, we obtained the explicit term for the state occupancy of the inhibitor bound state. We derived the expression for the IC_50_ of inhibitor displacement by substrate as follows: we first define the state occupancy of the inhibitor bound state with [S]o set to zero as the control condition. We then derived the expression for the IC_50_ for inhibitor displacement by the substrate by solving for the substrate concentration [S]o, at which the occupancy of the inhibitor bound state is half of the occupancy of the inhibitor bound state at the control condition. The explicit term for IC_50_ for inhibitor displacement by the substrate can be found in the supplement.

The rate of substrate release is given by the sum of the net fluxes through the cycles via which the substrate can leave the interior of the cell. As was shown in Figure 2a in the main text, there are two cycles which need to be considered: (1) the first cycle, which results in basal substrate release and (2) the second cycle, which involves cellular uptake of the releaser. We assumed that in the substrate release experiments the extracellular substrate concentration is zero. We therefore substituted in the resulting terms [S]o with zero. The explicit term for the rate of substrate release can be found in the supplement.

**Incorporation of voltage dependence**

Voltage dependence can be incorporated as follows:

(A4) $$k_{n}=k_{n}^{0}\cdot e^{\left(\frac{z_{n}\cdot V_{M}}{2\cdot R\cdot T\cdot F}\right)}$$

where *k_n_* is the *n_th_* rate, *z_n_* the associated valence, $k_{n}^{0}\;$the rate at zero millivolt, *R* the gas constant, *F* the Faraday constant, *T* the temperature in Kelvin, and V_M_ the membrane potential. For voltage independent rates, z_n_ is zero.
