# Supplementary material for: Descriptors of Secondary Active Transporter Function and How They Relate to Partial Reactions in the Transport Cycle
Source: Membranes (Basel). 2021 Mar 3;11(3):178. doi: 10.3390/membranes11030178 (PMC8001282; doi:10.3390/membranes11030178)
Supplement: Supplementary file 1 [file membranes-11-00178-s001.pdf]

membranes

MDPI

Supplementary File

Explicit terms for descriptors of transport function

Klaus Schicker [1], Shreyas Bhat [2], Clemens Farr [2], Verena Burtscher [2], Andreas Horner [3], Michael Freissmuth [2] and Walter Sandtner [2,*]

[1] Center for Physiology and Pharmacology, Division of Neurophysiology and Neuropharmacology, Medical University of Vienna, 1090 Vienna, Austria; klaus.schicker@meduniwien.ac.at
[2] Center of Physiology and Pharmacology, Institute of Pharmacology and the Gaston H. Glock Research Laboratories for Exploratory Drug Development, Medical University of Vienna, 1090 Vienna, Austria; shreyas.bhat@meduniwien.ac.at (S.B.); Clemens.Farr@meduniwien.ac.at (C.F.); verena.burtscher@meduniwien.ac.at (V.B.); michael.freissmuth@meduniwien.ac.at (M.F.)
[3] Institute of Biophysics, Johannes Kepler University Linz, 4040 Linz, Austria; Andreas.Horner@jku.at
* Correspondence: walter.sandtner@meduniwien.ac.at

V_{max} =

([Na]o*k34*k47*k78*k81*k120)/(k18*k34*k47*k78 + k34*k47*k78*k81 + [Na]o*k34*k47*k78*k120 + [Na]o * k34*k47*k81*k120 + [Na]o*k34*k78*k81*k120 + [Na]o*k43*k78*k81*k120 + [Na]o*k47*k78*k81*k120 + [Na]i* k18*k34*k47*k870 + [Na]i*[Na]o*k34*k47*k120*k870)

K_M =

((k32*k43 + k32*k47 + k34*k47)*(k18*k21*k78 + k21*k78*k81 + [Na]o*k78*k81*k120 + [Na]i *k18 *k21* k870))/ (k230*(k18*k34*k47*k78 + k34*k47*k78*k81 + [Na]o*k34*k47*k78*k120 + [Na]o*k34*k47*k81*k120 + [Na]o* k34*k78*k81*k120 + [Na]o*k43*k78*k81*k120 + [Na]o*k47*k78*k81*k120 + [Na]i*k18*k34*k47*k870 + [Na]i* [Na]o*k34*k47*k120*k870))

IC_{50} for displacement of an inhibitor by substrate =

(k18*k21*k78*k92* ([Na]o)*((k21*k32*k43*k78*k81^2*k92*k120*k740-
[Na]o*k34*k78^2*k81^2*k92* k120^2* k230-[Na]o*k43*k78^2*k81^2*k92*k120^2*k230-
[Na]o*k47*k78^2*k81^2*k92*k120^2*k230-k18* k34* k47* k78^2*k81*k92*k120*k230-
k34*k47*k78^2*k81^2*k92*k120*k230+2*k18^2* k21*k32*k43*k78* k92*k740* k870-
[Na]o*k34*k47*k78*k81^2*k92*k120^2*k230-[Na]o*k34*k47*k78^2*k81* k92*k120^2*
k230+ [Na]o*k32*
k43*k78*k81^2*k92*k120^2*k740+2*[Na]o*k18^2*k21*k32*k43*k92*k740*k870^2+k18*k21*
k32*k43*k78*k81*k92*k120*k740+2*k18*k21*k32*k43*k78*k81*k92*k740*k870+[I]o*[Na]o*
k32*k43*k78*k81^2*k120^2*k290*k740-[Na]o^2*k34*k47*k78*k81*k92*
k120^2*k230*k870+2*[Na]o^2*k18*k21*k32* k43*k92* k120*k740*
k870^2+[Na]o^2*k32*k43*k78*k81*k92*k120^2*k740*k870-[Na]o*k18*k34*k47*k78*k81*
k92* k120*k230*
k870+2*[Na]o*k18*k21*k32*k43*k78*k92*k120*k740*k870+2*[Na]o*k18*k21*k32*k43*k81*
k92*k120*k740*k870-[Na]o*k18*k21*k32*k78*k81*k92*k120*k740*k870-
[Na]o*k18*k21*k34*k78*k81* k92* k120*k740*k870-
[Na]o*k18*k21*k43*k78*k81*k92*k120*k740*k870+[Na]o* k18*k32*k43* k78*k81*k92*
k120*k740*k870+
[Na]o*k21*k32*k43*k78*k81*k92*k120*k740*k870+[I]o*[Na]o^2*k32*k43*k78*k81*k120^2*
k290*k740*k870+[I]o*[Na]o*k18*k32*k43*k78*k81*k120*k290*k740*k870)^2+4*[Na]o*k78^
2*k81^2*k92*k120^2*k230*k740*(k34+k43)*(k81*k120+k18*k870+[Na]o*k120*k870)*(k32*k
43+k32*k47+k34*k47)*(k18*k21*k78*k92+k21*k78*k81*k92+[Na]o*k78*k81*k92*k120+[Na]
o*k18*k21*k92*k870+[I]o*[Na]o*k78*k81*k120*k290))^(1/2)+k21*k78*k81*k92*
([Na]o)*((k21*k32*k43*k78*k81^2*k92*k120*k740-[Na]o*k34* k78^2*
k81^2*k92*k120^2*k230-[Na]o* k43*k78^2*k81^2*k92*k120^2*k230-
[Na]o*k47*k78^2*k81^2*k92* k120^2* k230-k18*k34* k47*k78^2*k81* k92*k120*k230-
k34*k47*k78^2*k81^2*k92*k120*k230+2* k18^2*k21* k32* k43*k78*k92*k740*k870-[Na]o*

k34*k47*k78*k81^2*k92*k120^2*k230-[Na]o*k34*k47*k78^2*k81*k92*
k120^2*k230+[Na]o*k32*k43* k78*
k81^2*k92*k120^2*k740+2*[Na]o*k18^2*k21*k32*k43*k92*k740*k870^2+k18*k21*k32*k43*
k78*k81*k92*k120*k740+2*k18*k21*k32*k43*k78*k81*k92*k740*k870+[I]o*[Na]o*k32*k43*
k78*k81^2*k120^2*k290*k740-[Na]o^2*k34*k47*k78*k81*k92*k120^2*k230*
k870+2*[Na]o^2*k18* k21*k32*k43*k92* k120*k740* k870^2+
[Na]o^2*k32*k43*k78*k81*k92*k120^2*k740*k870-[Na]o*k18*k34*
k47*k78*k81*k92*k120*k230*k870+2*
[Na]o*k18*k21*k32*k43*k78*k92*k120*k740*k870+2*[Na]o*k18*k21*k32*k43*k81*k92*k12
0*k740*k870-[Na]o*k18*k21*k32*k78*k81*k92*k120*k740*k870-[Na]o*k18*k21* k34*
k78*k81*k92*k120*k740*k870-
[Na]o*k18*k21*k43*k78*k81*k92*k120*k740*k870+[Na]o*k18* k32*k43*
k78*k81*k92*k120*k740* k870+
[Na]o*k21*k32*k43*k78*k81*k92*k120*k740*k870+[I]o*[Na]o^2*k32*k43*k78*k81*k120^2*
k290*k740*k870+[I]o*[Na]o*k18*k32*k43*k78*k81*k120*k290*k740*k870)^2+4*[Na]o*k78^
2*k81^2*k92*k120^2*k230*k740*(k34+k43)*(k81*k120+k18*k870+[Na]o*k120*k870)*(k32*k
43+k32*k47+k34*k47)*(k18*k21*k78*k92+k21*k78*k81*k92+[Na]o*k78*k81*k92*k120+[Na]
o*k18*k21*k92*k870+[I]o*[Na]o*k78*k81*k120*k290))^(1/2)+[Na]o*k78*k81*k92*k120*
([Na]o)*((k21*k32*k43*k78*k81^2*k92*k120*k740-
[Na]o*k34*k78^2*k81^2*k92*k120^2*k230-[Na]o*k43*k78^2*k81^2*k92*k120^2*k230-
[Na]o*k47*k78^2* k81^2*k92*k120^2*k230-k18*k34*k47*k78^2* k81*k92*k120*k230-
k34*k47*k78^2*k81^2*k92*k120*k230+ 2*k18^2*k21*k32*k43*k78*k92*k740*k870-
[Na]o*k34*k47*k78*k81^2*k92*k120^2*k230-[Na]o*k34*k47*
k78^2*k81*k92*k120^2*k230+ [Na]o*k32* k43*
k78*k81^2*k92*k120^2*k740+2*[Na]o*k18^2*k21*k32*k43*k92*k740*k870^2+k18*k21*k32*
k43*k78*k81*k92*k120*k740+2*k18*k21*k32*k43*k78*k81*k92*k740*k870+[I]o*[Na]o*k32*
k43*k78*k81^2*k120^2*k290*k740-[Na]o^2*k34*k47*k78*k81*k92*k120^2*k230* k870+2*
[Na]o^2*k18*k21*k32*k43*k92*k120*k740*k870^2+
[Na]o^2*k32*k43*k78*k81*k92*k120^2*k740*k870-[Na]o*
k18*k34*k47*k78*k81*k92*k120*k230*k870+2*
[Na]o*k18*k21*k32*k43*k78*k92*k120*k740*k870+2*[Na]o*k18*k21*k32*k43*k81*k92*k12
0*k740*k870-[Na]o*k18*k21*k32*k78*k81*k92*k120*k740*k870-[Na]o* k18*
k21*k34*k78*k81*k92*k120*k740*k870-[Na]o*k18*k21*k43 *k78*k81*k92*k120*k740*
k870+ [Na]o*k18* k32* k43*k78*k81*k92*k120*k740*k870+
[Na]o*k21*k32*k43*k78*k81*k92*k120*k740*k870+[I]o*[Na]o^2*k32*k43*k78*k81*k120^2*
k290*k740*k870+[I]o*[Na]o*k18*k32*k43*k78*k81*k120*k290*k740*k870)^2+4*[Na]o*k78^
2*k81^2*k92*k120^2*k230*k740*(k34+k43)*(k81*k120+k18*k870+[Na]o*k120*k870)*(k32*k
43+k32*k47+k34*k47)*(k18*k21*k78*k92+k21*k78*k81*k92+[Na]o*k78*k81*k92*k120+[Na]
o*k18*k21*k92*k870+[I]o*[Na]o*k78*k81*k120*k290))^(1/2)+[Na]o*k18*k21*k92*k870*
([Na]o)*((k21*k32*k43*k78*k81^2*k92*k120*k740-
[Na]o*k34*k78^2*k81^2*k92*k120^2*k230-[Na]o*k43*k78^2*k81^2*k92*k120^2*k230-
[Na]o* k47*k78^2* k81^2*k92*k120^2*k230-k18*k34*k47*k78^2* k81*k92*k120*k230-
k34*k47*k78^2* k81^2*k92*k120* k230+ 2*k18^2*k21*k32*k43*k78*k92*k740*k870-
[Na]o*k34*k47*k78*k81^2*k92*k120^2*k230-[Na]o*k34* k47*
k78^2*k81*k92*k120^2*k230+ [Na]o*k32*
k43*k78*k81^2*k92*k120^2*k740+2*[Na]o*k18^2*k21*k32*k43*k92*k740*k870^2+k18*k21*
k32*k43*k78*k81*k92*k120*k740+2*k18*k21*k32*k43*k78*k81*k92*k740*k870+[I]o*[Na]o*
k32*k43*k78*k81^2*k120^2*k290*k740-[Na]o^2*k34*k47*k78*k81*k92*k120^2*k230*
k870+ 2* [Na]o^2*k18*k21*k32*k43*k92*k120*k740*
k870^2+[Na]o^2*k32*k43*k78*k81*k92*k120^2* k740*k870-
[Na]o*k18*k34*k47*k78*k81*k92*k120*k230*
k870+2*[Na]o*k18*k21*k32*k43*k78*k92*k120*k740*k870+2*[Na]o*k18*k21*k32*k43*k81*
k92*k120*k740*k870-[Na]o*k18*k21*k32*k78*k81*k92*k120*k740*k870-[Na]o*
k18*k21*k34*k78*k81*k92*k120*k740*k870-

[Na]o*k18*k21*k43*k78*k81*k92*k120*k740*k870+[Na]o*k18*
k32*k43*k78*k81*k92*k120*k740*k870+
[Na]o*k21*k32*k43*k78*k81*k92*k120*k740*k870+[I]o*[Na]o^2*k32*k43*k78*k81*k120^2*
k290*k740*k870+[I]o*[Na]o*k18*k32*k43*k78*k81*k120*k290*k740*k870)^2+4*[Na]o*k78^
2*k81^2*k92*k120^2*k230*k740*(k34+k43)*(k81*k120+k18*k870+[Na]o*k120*k870)*(k32*k
43+k32*k47+k34*k47)*(k18*k21*k78*k92+k21*k78*k81*k92+[Na]o*k78*k81*k92*k120+[Na]
o*k18*k21*k92*k870+[I]o*[Na]o*k78*k81*k120*k290))^(1/2)+[I]o*[Na]o*k78*k81*k120*k29
0* ([Na]o)*((k21*k32*k43*k78*k81^2*k92*k120*k740-[Na]o*k34*k78^2*k81^2*k92*k120^2*
k230-[Na]o*k43*k78^2*k81^2*k92*k120^2*k230-[Na]o*k47*
k78^2*k81^2*k92*k120^2*k230-k18*k34*k47* k78^2*k81*k92*k120*k230-
k34*k47*k78^2*k81^2* k92*k120* k230+2*k18^2*k21*k32*k43*k78*k92* k740* k870-
[Na]o*k34*k47*k78*k81^2*k92*k120^2*k230-[Na]o*k34*
k47*k78^2*k81*k92*k120^2*k230+ [Na]o*k32*
k43*k78*k81^2*k92*k120^2*k740+2*[Na]o*k18^2*k21*k32*k43*k92*k740*k870^2+k18*k21*
k32*k43*k78*k81*k92*k120*k740+2*k18*k21*k32*k43*k78*k81*k92*k740*k870+[I]o*[Na]o*
k32*k43*k78*k81^2*k120^2*k290*k740-
[Na]o^2*k34*k47*k78*k81*k92*k120^2*k230*k870+2* [Na]o^2*k18*k21*k32*k43*k92*
k120*k740* k870^2+[Na]o^2*k32*k43*k78*k81*k92*k120^2*k740*k870-
[Na]o*k18*k34*k47*k78*k81* k92*k120* k230*
k870+2*[Na]o*k18*k21*k32*k43*k78*k92*k120*k740*k870+2*[Na]o*k18*k21*k32*k43*k81*
k92*k120*k740*k870-[Na]o*k18*k21*k32*k78*k81*k92*k120*k740*k870-[Na]o*
k18*k21*k34*k78*k81*k92*k120*k740*k870-
[Na]o*k18*k21*k43*k78*k81*k92*k120*k740*k870+[Na]o* k18*
k32*k43*k78*k81*k92*k120*k740* k870+
[Na]o*k21*k32*k43*k78*k81*k92*k120*k740*k870+[I]o*[Na]o^2*k32*k43*k78*k81*k120^2*
k290*k740*k870+[I]o*[Na]o*k18*k32*k43*k78*k81*k120*k290*k740*k870)^2+4*[Na]o*k78^
2*k81^2*k92*k120^2*k230*k740*(k34+k43)*(k81*k120+k18*k870+[Na]o*k120*k870)*(k32*k
43+k32*k47+k34*k47)*(k18*k21*k78*k92+k21*k78*k81*k92+[Na]o*k78*k81*k92*k120+[Na]
o*k18*k21*k92*k870+[I]o*[Na]o*k78*k81*k120*k290))^(1/2)-[Na]o^2*
k34*k78^2*k81^2*k92*k120^2*k230* (k18*k21*k78*k92+k21*k78* k81* k92+
[Na]o*k78*k81*k92*k120 +[Na]o*k18*k21*k92*k870+[I]o*[Na]o*k78*k81*k120*k290)-
[Na]o^2* k43* k78^2*k81^2*k92*k120^2*k230*
(k18*k21*k78*k92+k21*k78*k81*k92+[Na]o*k78*k81*k92*k120+[Na]o*k18*k21*k92*k870+[
I]o*[Na]o*k78*k81*k120*k290)-[Na]o^2*k47*k78^2*k81^2*k92*k120^2*k230*
(k18*k21*k78*k92+k21*k78*k81*k92+ [Na]o* k78*
k81*k92*k120+[Na]o*k18*k21*k92*k870+[I]o*[Na]o*k78*k81*k120*k290)-
[Na]o*k34*k47*k78^2*k81^2* k92* k120*k230*
(k18*k21*k78*k92+k21*k78*k81*k92+[Na]o*k78*k81*k92*k120+[Na]o*k18*k21*k92* k870+
[I]o* [Na]o*k78*k81*k120*k290)-[Na]o^2*k34*k47*k78*k81^2*k92*k120^2*k230*
(k18*k21*k78*k92+k21*k78*k81*
k92+[Na]o*k78*k81*k92*k120+[Na]o*k18*k21*k92*k870+[I]o*[Na]o*k78*k81*k120*k290)-
[Na]o^2*k34*k47* k78^2*k81*k92*k120^2*k230*
(k18*k21*k78*k92+k21*k78*k81*k92+[Na]o*k78*k81* k92*k120+[Na]o* k18*
k21*k92*k870+[I]o*[Na]o*k78*k81*k120*k290)+[Na]o^2*k32*k43*k78*k81^2*k92*k120^2*k
740* (k18*k21*
k78*k92+k21*k78*k81*k92+[Na]o*k78*k81*k92*k120+[Na]o*k18*k21*k92*k870+[I]o*[Na]o
*k78*k81*k120*k290)+2*[Na]o^2*k18^2*k21*k32*k43*k92*k740*k870^2*
(k18*k21*k78*k92+k21*k78*k81* k92+[Na]o*k78*k81*
k92*k120+[Na]o*k18*k21*k92*k870+[I]o*[Na]o*k78*k81*k120*k290)-
[Na]o*k18*k34*k47*k78^2*k81*k92* k120*k230*
(k18*k21*k78*k92+k21*k78*k81*k92+[Na]o*k78*k81*k92*k120+[Na]o* k18*k21*k92*
k870+[I]o* [Na]o* k78*k81*k120*k290)+[Na]o*k21*k32*k43*k78*k81^2*k92*k120*k740*
(k18*k21*k78*k92+k21* k78*
k81*k92+[Na]o*k78*k81*k92*k120+[Na]o*k18*k21*k92*k870+[I]o*[Na]o*k78*k81*k120*k2

90)+2*[Na]o*k18^2*k21*k32*k43*k78*k92*k740*k870*
(k18*k21*k78*k92+k21*k78*k81*k92+ [Na]o*k78*k81* k92*k120+[Na]o*
k18*k21*k92*k870+[I]o*[Na]o*k78*k81*k120*k290)-
[Na]o^3*k34*k47*k78*k81*k92*k120^2*k230*k870* (k18*
k21*k78*k92+k21*k78*k81*k92+[Na]o*k78*k81*k92*k120+[Na]o*k18*k21*k92*k870+[I]o*[
Na]o*k78*k81*k120*k290)+2*[Na]o^3*k18*k21*k32*k43*k92*k120*k740*k870^2*
(k18*k21*k78*k92+k21*k78*k81* k92+[Na]o*
k78*k81*k92*k120+[Na]o*k18*k21*k92*k870+[I]o*[Na]o*k78*k81*k120*k290)+[Na]o^3*k3
2*k43*k78*k81*k92*k120^2*k740*k870*
(k18*k21*k78*k92+k21*k78*k81*k92+[Na]o*k78*k81*k92* k120+[Na]o*k18*k21*k92*
k870+[I]o*[Na]o*k78*k81*k120*k290)+[I]o*[Na]o^2*k32*k43*k78*k81^2*k120^2*k290*k740
* (k18*k21*k78*
k92+k21*k78*k81*k92+[Na]o*k78*k81*k92*k120+[Na]o*k18*k21*k92*k870+[I]o*[Na]o*k78
*k81*k120*k290)+[I]o*[Na]o^3*k32*k43*k78*k81*k120^2*k290*k740*k870*
(k18*k21*k78*k92+k21*k78* k81*k92+[Na]o*k78*
k81*k92*k120+[Na]o*k18*k21*k92*k870+[I]o*[Na]o*k78*k81*k120*k290)+[Na]o*k18*k21*k
32*k43*k78*k81*k92*k120*k740* (k18*k21*k78*k92+k21*k78*k81*k92+[Na]o*k78*k81*k92*
k120+[Na]o*k18* k21*k92*k870+
[I]o*[Na]o*k78*k81*k120*k290)+2*[Na]o*k18*k21*k32*k43*k78*k81*k92*k740*k870*
(k18*k21*k78* k92+ k21*
k78*k81*k92+[Na]o*k78*k81*k92*k120+[Na]o*k18*k21*k92*k870+[I]o*[Na]o*k78*k81*k12
0*k290)-[Na]o^2*k18*k34*k47*k78*k81*k92*k120*k230*k870*
(k18*k21*k78*k92+k21*k78*k81* k92+[Na]o*k78* k81*
k92*k120+[Na]o*k18*k21*k92*k870+[I]o*[Na]o*k78*k81*k120*k290)+2*[Na]o^2*k18*k21*k
32*k43*k78*k92*k120*k740*k870*
(k18*k21*k78*k92+k21*k78*k81*k92+[Na]o*k78*k81*k92*k120+ [Na]o*k18*k21* k92*k870+
[I]o*[Na]o*k78*k81*k120*k290)+2*[Na]o^2*k18*k21*k32*k43*k81*k92*k120*k740*k870*
(k18*k21*k78*k92+
k21*k78*k81*k92+[Na]o*k78*k81*k92*k120+[Na]o*k18*k21*k92*k870+[I]o*[Na]o*k78*k81
*k120*k290)-[Na]o^2*k18*k21*k32*k78*k81*k92*k120*k740*k870*
(k18*k21*k78*k92+k21*k78*k81*k92+[Na]o*k78*k81 *
k92*k120+[Na]o*k18*k21*k92*k870+[I]o*[Na]o*k78*k81*k120*k290)-
[Na]o^2*k18*k21*k34*k78*k81* k92* k120*k740*k870*
(k18*k21*k78*k92+k21*k78*k81*k92+[Na]o*k78*k81*k92*k120+ [Na]o*k18*k21*k92*k870+
[I]o*[Na]o*k78*k81*k120*k290)-[Na]o^2*k18*k21*k43*k78*k81*k92*k120*k740*k870*
(k18*k21*k78*k92+
k21*k78*k81*k92+[Na]o*k78*k81*k92*k120+[Na]o*k18*k21*k92*k870+[I]o*[Na]o*k78*k81
*k120*k290)+[Na]o^2*k18*k32*k43*k78*k81*k92*k120*k740*k870*
(k18*k21*k78*k92+k21*k78*k81*k92+[Na]o*k78*k81*k92*
k120+[Na]o*k18*k21*k92*k870+[I]o*[Na]o*k78*k81*k120*k290)+[Na]o^2*k21*k32*k43*k7
8*k81*k92*k120*k740*k870*
(k18*k21*k78*k92+k21*k78*k81*k92+[Na]o*k78*k81*k92*k120+[Na]o*k18* k21*k92*k870+
[I]o*
[Na]o*k78*k81*k120*k290)+[I]o*[Na]o^2*k18*k32*k43*k78*k81*k120*k290*k740*k870*
(k18*k21*k78*k92+
k21*k78*k81*k92+[Na]o*k78*k81*k92*k120+[Na]o*k18*k21*k92*k870+[I]o*[Na]o*k78*k81
*k120*k290))/(2*[Na]o^2*k78*k81*k92*k120*k230*k740*
(k18*k21*k78*k92+k21*k78*k81*k92+ [Na]o*k78*k81* k92*k120+[Na]o*
18*k21*k92*k870+[I]o*[Na]o*k78*k81*k120*k290)*(k34+k43)*(k81*k120+k18*k870+[Na]o*
k120*k870))

Rate of release =

([S]i*k32*k43*k740*([Na]i*k18*k21*k52*k65*k870+[Na]i*k18*k21*k52*k67*k870+[Na]
i*k18*k21*k56*k67*k870+[Na]o*[R]o*k56*k67*k81*k120*k250+[Na]i*[R]o*k18*k56*k67*k2
50*k870+[Na]i*[Na]o*[R]o*k56*k67*k120*k250*k870))/(k18*k21*k32*k43*k52*k65*k78+k18

*k21*k32*k43*k52*k67*k78+k18*k21*k32*k47*k52*k65*k78+k18*k21*k32*k43*k56*k67*k78
+k18*k21*k32*k47*k52*k67*k78+k18*k21*k34*k47*k52*k65*k78+k18*k21*k34*k47*k52*k67
*k78+k18*k21*k32*k47*k56*k67*k78+k18*k21*k34*k47*k56*k67*k78+k21*k32*k43*k52*k65
*k78*k81+k21*k32*k43*k52*k67*k78*k81+k21*k32*k47*k52*k65*k78*k81+k21*k32*k43*k56
*k67*k78*k81+k21*k32*k47*k52*k67*k78*k81+k21*k34*k47*k52*k65*k78*k81+k21*k34*k47
*k52*k67*k78*k81+k21*k32*k47*k56*k67*k78*k81+k21*k34*k47*k56*k67*k78*k81+[Na]o*k
32*k43*k52*k65*k78*k81*k120+[Na]o*k32*k43*k52*k67*k78*k81*k120+[Na]o*k32*k47*k52
*k65*k78*k81*k120+[Na]o*k32*k43*k56*k67*k78*k81*k120+[Na]o*k32*k47*k52*k67*k78*k
81*k120+[Na]o*k34*k47*k52*k65*k78*k81*k120+[Na]o*k34*k47*k52*k67*k78*k81*k120+[
Na]o*k32*k47*k56*k67*k78*k81*k120+[Na]o*k34*k47*k56*k67*k78*k81*k120+[Na]i*k18*k
21*k32*k43*k52*k65*k870+[Na]i*k18*k21*k32*k43*k52*k67*k870+[Na]i*k18*k21*k32*k47*
k52*k65*k870+[Na]i*k18*k21*k32*k43*k56*k67*k870+[Na]i*k18*k21*k32*k47*k52*k67*k87
0+[Na]i*k18*k21*k34*k47*k52*k65*k870+[Na]i*k18*k21*k34*k47*k52*k67*k870+[Na]i*k18
*k21*k32*k47*k56*k67*k870+[Na]i*k18*k21*k34*k47*k56*k67*k870+[R]o*k18*k32*k43*k56
*k67*k78*k250+[R]o*k18*k32*k47*k56*k67*k78*k250+[R]o*k18*k34*k47*k56*k67*k78*k25
0+[R]o*k32*k43*k56*k67*k78*k81*k250+[R]o*k32*k47*k56*k67*k78*k81*k250+[R]o*k34*k4
7*k56*k67*k78*k81*k250+[S]i*k18*k21*k32*k43*k52*k65*k740+[S]i*k18*k21*k32*k43*k52*
k67*k740+[S]i*k18*k21*k32*k43*k56*k67*k740+[S]i*k21*k32*k43*k52*k65*k81*k740+[S]i*k
21*k32*k43*k52*k67*k81*k740+[S]i*k21*k32*k43*k56*k67*k81*k740+[Na]o*[R]o*k32*k43*
k56*k67*k78*k120*k250+[Na]o*[R]o*k32*k43*k56*k67*k81*k120*k250+[Na]o*[R]o*k32*k4
7*k56*k67*k78*k120*k250+[Na]o*[R]o*k34*k47*k56*k67*k78*k120*k250+[Na]o*[R]o*k32*
k47*k56*k67*k81*k120*k250+[Na]o*[R]o*k34*k47*k56*k67*k81*k120*k250+[Na]o*[R]o*k3
2*k43*k56*k78*k81*k120*k250+[Na]o*[R]o*k32*k47*k56*k78*k81*k120*k250+[Na]o*[R]o*
k34*k47*k56*k78*k81*k120*k250+[Na]o*[R]o*k32*k43*k65*k78*k81*k120*k250+[Na]o*[R]
o*k32*k43*k67*k78*k81*k120*k250+[Na]o*[R]o*k32*k47*k65*k78*k81*k120*k250+[Na]o*[
R]o*k32*k47*k67*k78*k81*k120*k250+[Na]o*[R]o*k34*k47*k65*k78*k81*k120*k250+[Na]o
*[R]o*k34*k47*k67*k78*k81*k120*k250+[Na]i*[R]o*k18*k32*k43*k56*k67*k250*k870+[Na]
i*[R]o*k18*k32*k47*k56*k67*k250*k870+[Na]i*[R]o*k18*k34*k47*k56*k67*k250*k870+[Na
]o*[S]i*k32*k43*k52*k65*k81*k120*k740+[Na]o*[S]i*k32*k43*k52*k67*k81*k120*k740+[Na
]o*[S]i*k32*k43*k56*k67*k81*k120*k740+[Na]i*[S]i*k18*k21*k32*k52*k65*k740*k870+[Na]
i*[S]i*k18*k21*k32*k52*k67*k740*k870+[Na]i*[S]i*k18*k21*k34*k52*k65*k740*k870+[Na]i*
[S]i*k18*k21*k34*k52*k67*k740*k870+[Na]i*[S]i*k18*k21*k32*k56*k67*k740*k870+[Na]i*[
S]i*k18*k21*k34*k56*k67*k740*k870+[Na]i*[S]i*k18*k21*k43*k52*k65*k740*k870+[Na]i*[S
]i*k18*k21*k43*k52*k67*k740*k870+[Na]i*[S]i*k18*k21*k43*k56*k67*k740*k870+[Na]i*[S]i
*k18*k32*k43*k52*k65*k740*k870+[Na]i*[S]i*k18*k32*k43*k52*k67*k740*k870+[Na]i*[S]i*
k21*k32*k43*k52*k65*k740*k870+[Na]i*[S]i*k21*k32*k43*k52*k67*k740*k870+[Na]i*[S]i*k
18*k32*k43*k56*k67*k740*k870+[Na]i*[S]i*k21*k32*k43*k56*k67*k740*k870+[Na]i*[Na]o*
[R]o*k32*k43*k56*k67*k120*k250*k870+[Na]i*[Na]o*[R]o*k32*k47*k56*k67*k120*k250*k8
70+[Na]i*[Na]o*[R]o*k34*k47*k56*k67*k120*k250*k870+[Na]i*[Na]o*[S]i*k32*k43*k52*k6
5*k120*k740*k870+[Na]i*[Na]o*[S]i*k32*k43*k52*k67*k120*k740*k870+[Na]i*[Na]o*[S]i*k
32*k43*k56*k67*k120*k740*k870+[Na]o*[R]o*[S]i*k32*k43*k56*k81*k120*k250*k740+[Na]
o*[R]o*[S]i*k32*k43*k65*k81*k120*k250*k740+[Na]o*[R]o*[S]i*k32*k43*k67*k81*k120*k25
0*k740+[Na]o*[R]o*[S]i*k32*k56*k67*k81*k120*k250*k740+[Na]o*[R]o*[S]i*k34*k56*k67*k
81*k120*k250*k740+[Na]o*[R]o*[S]i*k43*k56*k67*k81*k120*k250*k740+[Na]i*[R]o*[S]i*k1
8*k32*k43*k56*k250*k740*k870+[Na]i*[R]o*[S]i*k18*k32*k43*k65*k250*k740*k870+[Na]i*[
R]o*[S]i*k18*k32*k43*k67*k250*k740*k870+[Na]i*[R]o*[S]i*k18*k32*k56*k67*k250*k740*k
870+[Na]i*[R]o*[S]i*k18*k34*k56*k67*k250*k740*k870+[Na]i*[R]o*[S]i*k18*k43*k56*k67*k
250*k740*k870+[Na]i*[Na]o*[R]o*[S]i*k32*k43*k56*k120*k250*k740*k870+[Na]i*[Na]o*[R
]o*[S]i*k32*k43*k65*k120*k250*k740*k870+[Na]i*[Na]o*[R]o*[S]i*k32*k43*k67*k120*k250
*k740*k870+[Na]i*[Na]o*[R]o*[S]i*k32*k56*k67*k120*k250*k740*k870+[Na]i*[Na]o*[R]o*[
S]i*k34*k56*k67*k120*k250*k740*k870+[Na]i*[Na]o*[R]o*[S]i*k43*k56*k67*k120*k250*k7
40*k870)

The expression below gives the ratio of substrate release between mutant and wild-type in the reparameterized formulation. If this term assumes values larger than 1 the

mutant releases more substrate than wild-type. We evaluated this expression with the *fmincon* function as described in the main manuscript.

Release$_{Mut}$/Release$_{wt}$ =

(X*([Na]i*F_ES*F_NK*F_RK*k21*k34+[Na]i*F_ES*F_NK*F_RK*k21*k52+[Na]i*[Na]o
*[R]o*F_NK*F_RK*k120*k250+[Na]i*[R]o*F_ES*F_NK*F_RK*k34*k250+[Na]i*F_ES*F_IO*
F_NK*X*k21*k34+[Na]o*[R]o*F_ES*F_IO*F_RK*X*k34*k250)*(F_ES*F_NK*F_RK*F_SK*k
21^2*k32^2*k52^2+F_ES*F_IO^2*F_NK*k21^2*k32*k34^2*k52+F_ESF_IO^3*F_NK*k21^2*
k32*k34^2*k52+F_ES*F_IO*F_NK*F_RK*F_SK*k21^2*k32^2*k52^2+F_ES*F_IO^2*F_NK*F
_RK*k21^2*k32*k34*k52^2+F_ES*F_IO^2*F_NK*F_RK*k21^2*k32*k34^2*k52+F_ES*F_IO
^2*F_NK*F_SK*k21^2*k32*k34^2*k52+F_ES*F_IO^2*F_NK*F_SK*k21^2*k32^2*k34*k52+F
_ES*F_IO*F_NK*F_RK*k21^2*k32*k34*k52^2+F_ES*F_IO*F_NK*F_RK*k21^2*k32*k34^2*
k52+F_ES*F_IO*F_NK*F_SK*k21^2*k32*k34^2*k52+F_ES*F_IO*F_NK*F_SK*k21^2*k32^2
*k34*k52+F_ES*F_NK*F_RK*F_SK*k21^2*k32*k34*k52^2+F_ES*F_NK*F_RK*F_SK*k21^2
*k32*k34^2*k52+F_ES*F_NK*F_RK*F_SK*k21^2*k32^2*k34*k52+[Na]i*F_ES*F_NK*F_RK
*F_SK*k21*k32^2*k52^2*k120+F_ES*F_IO*F_NK*F_RK*F_SK*k21^2*k32*k34*k52^2+F_ES
*F_IO*F_NK*F_RK*F_SK*k21^2*k32*k34^2*k52+F_ES*F_IO*F_NK*F_RK*F_SK*k21^2*k3
2^2*k34*k52+[Na]i*F_ES*F_IO^2*F_NK*k21*k32*k34^2*k52*k120+[Na]o*F_ES*F_IO^3*F_
NK*k21*k32*k34^2*k52*k120+[S]I*F_ES*F_IO^2*F_SK*k21*k32*k34^2*k52*k230+[S]I*F_E
S*F_IO^3*F_SK*k21*k32*k34^2*k52*k230+[Na]o*[R]o*F_NK*F_RK*F_SK*k21*k32^2*k52*
k120*k250+[Na]i*F_ES*F_IO*F_NK*F_RK*k21*k32*k34*k52^2*k120+[Na]i*F_ES*F_IO*F_
NK*F_RK*k21*k32*k34^2*k52*k120+[Na]i*F_ES*F_IO*F_NK*F_SK*k21*k32*k34^2*k52*k
120+[Na]i*F_ES*F_IO*F_NK*F_SK*k21*k32^2*k34*k52*k120+[Na]i*F_ES*F_NK*F_RK*F_
SK*k21*k32*k34*k52^2*k120+[Na]i*F_ES*F_NK*F_RK*F_SK*k21*k32*k34^2*k52*k120+[N
a]i*F_ES*F_NK*F_RK*F_SK*k21*k32^2*k34*k52*k120+[R]o*F_ES*F_IO*F_NK*F_RK*k21*
k32*k34^2*k52*k250+[R]o*F_ES*F_NK*F_RK*F_SK*k21*k32*k34^2*k52*k250+[R]o*F_ES*
F_NK*F_RK*F_SK*k21*k32^2*k34*k52*k250+[S]I*F_ES*F_IO*F_RK*F_SK*k21*k32*k34*k
52^2*k230+[S]I*F_ES*F_IO*F_RK*F_SK*k21*k32*k34^2*k52*k230+[Na]i*[Na]o*[R]o*F_N
K*F_RK*F_SK*k32^2*k52*k120^2*k250+[Na]o*F_ES*F_IO*F_NK*F_RK*F_SK*k21*k32^2*
k52^2*k120+[Na]o*[R]o*F_ES*F_IO^2*F_NK*k21*k32*k34^2*k120*k250+[Na]o*[R]o*F_ES
*F_IO^3*F_NK*k21*k32*k34^2*k120*k250+[Na]o*[R]o*F_ES*F_IO^2*F_RK*k32*k34^2*k5
2*k120*k250+[Na]o*[S]I*F_ES*F_IO^3*F_SK*k32*k34^2*k52*k120*k230+[Na]o*F_ES*F_IO
^2*F_NK*F_RK*k21*k32*k34*k52^2*k120+[Na]o*F_ES*F_IO^2*F_NK*F_RK*k21*k32*k34
^2*k52*k120+[Na]o*F_ES*F_IO^2*F_NK*F_SK*k21*k32*k34^2*k52*k120+[Na]o*F_ES*F_I
O^2*F_NK*F_SK*k21*k32^2*k34*k52*k120+[R]o*F_ES*F_IO^2*F_NK*F_RK*k21*k32*k34
^2*k52*k250+[S]I*F_ES*F_IO^2*F_RK*F_SK*k21*k32*k34*k52^2*k230+[S]I*F_ES*F_IO^2*
F_RK*F_SK*k21*k32*k34^2*k52*k230+[Na]o*[R]o*F_IO*F_NK*F_RK*k21*k32*k34*k52*k
120*k250+[Na]o*[R]o*F_NK*F_RK*F_SK*k21*k32*k34*k52*k120*k250+[Na]i*[Na]o*[R]o*
F_IO*F_NK*F_RK*k32*k34*k52*k120^2*k250+[Na]i*[Na]o*[R]o*F_NK*F_RK*F_SK*k32*k
34*k52*k120^2*k250+[Na]i*[R]o*F_ES*F_IO*F_NK*F_RK*k32*k34^2*k52*k120*k250+[Na]
o*[R]o*F_ES*F_IO*F_NK*F_SK*k21*k32*k34^2*k120*k250+[Na]o*[R]o*F_ES*F_IO*F_NK*
F_SK*k21*k32^2*k34*k120*k250+[Na]o*[R]o*F_ES*F_IO*F_RK*F_SK*k32*k34^2*k52*k12
0*k250+[Na]o*[R]o*F_ES*F_IO*F_RK*F_SK*k32^2*k34*k52*k120*k250+[Na]i*[R]o*F_ES*F
_NK*F_RK*F_SK*k32*k34^2*k52*k120*k250+[Na]i*[R]o*F_ES*F_NK*F_RK*F_SK*k32^2*
k34*k52*k120*k250+[Na]i*[S]I*F_ES*F_IO*F_NK*F_SK*k21*k34^2*k52*k120*k230+[Na]i*[
S]I*F_ES*F_NK*F_RK*F_SK*k21*k32*k52^2*k120*k230+[Na]i*[S]I*F_ES*F_NK*F_RK*F_S
K*k21*k34*k52^2*k120*k230+[Na]i*[S]I*F_ES*F_NK*F_RK*F_SK*k21*k34^2*k52*k120*k2
30+[Na]i*[S]I*F_IO*F_NK*F_RK*F_SK*k21*k32*k52^2*k120*k230+[Na]o*F_ES*F_IO*F_N
K*F_RK*F_SK*k21*k32*k34*k52^2*k120+[Na]o*F_ES*F_IO*F_NK*F_RK*F_SK*k21*k32*k
34^2*k52*k120+[Na]o*F_ES*F_IO*F_NK*F_RK*F_SK*k21*k32^2*k34*k52*k120+[R]o*F_E
S*F_IO*F_NK*F_RK*F_SK*k21*k32*k34^2*k52*k250+[R]o*F_ES*F_IO*F_NK*F_RK*F_SK
*k21*k32^2*k34*k52*k250+[Na]i*[S]I*F_IO^2*F_NK*F_SK*k21*k32*k34*k52*k120*k230+[
Na]i*[Na]o*[S]I*F_IO*F_NK*F_RK*F_SK*k32*k52^2*k120^2*k230+[Na]i*[Na]o*[S]I*F_IO
^2*F_NK*F_SK*k32*k34*k52*k120^2*k230+[Na]o*[R]o*[S]I*F_ES*F_IO^2*F_SK*k32*k34^
2*k120*k230*k250+[Na]o*[R]o*[S]I*F_ES*F_IO^3*F_SK*k32*k34^2*k120*k230*k250+[Na]o

*[R]o*F_ES*F_IO^2*F_NK*F_SK*k21*k32*k34^2*k120*k250+[Na]o*[R]o*F_ES*F_IO^2*F_
NK*F_SK*k21*k32^2*k34*k120*k250+[Na]i*[S]I*F_ES*F_IO^2*F_NK*F_SK*k21*k34^2*k5
2*k120*k230+[Na]i*[S]I*F_ES*F_IO^2*F_NK*F_SK*k32*k34^2*k52*k120*k230+[Na]o*[S]I*
F_ES*F_IO^2*F_RK*F_SK*k32*k34*k52^2*k120*k230+[Na]o*[S]I*F_ES*F_IO^2*F_RK*F_S
K*k32*k34^2*k52*k120*k230+[Na]i*[Na]o*[R]o*[S]I*F_IO^2*F_NK*F_SK*k32*k34*k120^2
*k230*k250+[Na]i*[R]o*[S]I*F_ES*F_IO^2*F_NK*F_SK*k32*k34^2*k120*k230*k250+[Na]o
[R]o[S]I*F_ES*F_IO^2*F_RK*F_SK*k34^2*k52*k120*k230*k250+[Na]i*[S]I*F_ES*F_IO*F
_NK*F_SK*k21*k32*k34*k52*k120*k230+[Na]i*[S]I*F_ES*F_NK*F_RK*F_SK*k21*k32*k34
*k52*k120*k230+[Na]i*[S]I*F_IO*F_NK*F_RK*F_SK*k21*k32*k34*k52*k120*k230+[Na]i*[
Na]o*[R]o*[S]I*F_IO*F_NK*F_SK*k32*k34*k120^2*k230*k250+[Na]i*[Na]o*[R]o*[S]I*F_N
K*F_RK*F_SK*k32*k52*k120^2*k230*k250+[Na]i*[Na]o*[R]o*[S]I*F_NK*F_RK*F_SK*k34*
k52*k120^2*k230*k250+[Na]i*[Na]o*[S]I*F_IO*F_NK*F_RK*F_SK*k32*k34*k52*k120^2*k
230+[Na]i*[R]o*[S]I*F_ES*F_IO*F_NK*F_SK*k32*k34^2*k120*k230*k250+[Na]o*[R]o*[S]I*
F_ES*F_IO*F_RK*F_SK*k34^2*k52*k120*k230*k250+[Na]i*[R]o*[S]I*F_ES*F_NK*F_RK*F
_SK*k34^2*k52*k120*k230*k250+[Na]o*[R]o*F_ES*F_IO*F_NK*F_RK*F_SK*k21*k32^2*k
52*k120*k250+[Na]i*[S]I*F_ES*F_IO*F_NK*F_RK*F_SK*k21*k34*k52^2*k120*k230+[Na]i*
[S]I*F_ES*F_IO*F_NK*F_RK*F_SK*k21*k34^2*k52*k120*k230+[Na]i*[S]I*F_ES*F_IO*F_N
K*F_RK*F_SK*k32*k34*k52^2*k120*k230+[Na]i*[S]I*F_ES*F_IO*F_NK*F_RK*F_SK*k32*k
34^2*k52*k120*k230+[Na]o*[R]o*F_ES*F_IO^2*F_NK*F_RK*k21*k32*k34*k52*k120*k250
+[Na]o*[R]o*[S]I*F_ES*F_IO^2*F_RK*F_SK*k32*k34*k52*k120*k230*k250+[Na]o*[R]o*[S]
I*F_ES*F_IO*F_RK*F_SK*k32*k34*k52*k120*k230*k250+[Na]i*[R]o*[S]I*F_ES*F_NK*F_R
K*F_SK*k32*k34*k52*k120*k230*k250+[Na]o*[R]o*F_ES*F_IO*F_NK*F_RK*F_SK*k21*k3
2*k34*k52*k120*k250+[Na]i*[Na]o*[R]o*[S]I*F_IO*F_NK*F_RK*F_SK*k32*k52*k120^2*k2
30*k250+[Na]i*[Na]o*[R]o*[S]I*F_IO*F_NK*F_RK*F_SK*k34*k52*k120^2*k230*k250+[Na]
i*[R]o*[S]I*F_ES*F_IO*F_NK*F_RK*F_SK*k34^2*k52*k120*k230*k250+[Na]i*[R]o*[S]I*F_
ES*F_IO*F_NK*F_RK*F_SK*k32*k34*k52*k120*k230*k250))/((([Na]i*F_ES*F_IO*F_NK*k2
1*k34+[Na]i*F_ES*F_NK*F_RK*k21*k34+[Na]i*F_ES*F_NK*F_RK*k21*k52+[Na]i*[Na]o*[
R]o*F_NK*F_RK*k120*k250+[Na]o*[R]o*F_ES*F_IO*F_RK*k34*k250+[Na]i*[R]o*F_ES*F_
NK*F_RK*k34*k250)*(F_ES*F_NK*F_RK*F_SK*k21^2*k32^2*k52^2+F_ES*F_IO^2*F_NK*
X^2*k21^2*k32*k34^2*k52+F_ES*F_IO^3*F_NK*X^3*k21^2*k32*k34^2*k52+F_ES*F_NK*F
_RK*F_SK*k21^2*k32*k34*k52^2+F_ES*F_NK*F_RK*F_SK*k21^2*k32*k34^2*k52+F_ES*F
_NK*F_RK*F_SK*k21^2*k32^2*k34*k52+[Na]i*F_ES*F_NK*F_RK*F_SK*k21*k32^2*k52^2
*k120+F_ES*F_IO*F_NK*F_RK*X*k21^2*k32*k34*k52^2+F_ES*F_IO*F_NK*F_RK*X*k21^
2*k32*k34^2*k52+F_ES*F_IO*F_NK*F_SK*X*k21^2*k32*k34^2*k52+F_ES*F_IO*F_NK*F_
SK*X*k21^2*k32^2*k34*k52+F_ES*F_IO*F_NK*F_RK*F_SK*X*k21^2*k32^2*k52^2+F_ES*
F_IO^2*F_NK*F_RK*X^2*k21^2*k32*k34*k52^2+F_ES*F_IO^2*F_NK*F_RK*X^2*k21^2*k
32*k34^2*k52+F_ES*F_IO^2*F_NK*F_SK*X^2*k21^2*k32*k34^2*k52+F_ES*F_IO^2*F_NK
*F_SK*X^2*k21^2*k32^2*k34*k52+[Na]i*F_ES*F_IO^2*F_NK*X^2*k21*k32*k34^2*k52*k12
0+[Na]o*F_ES*F_IO^3*F_NK*X^3*k21*k32*k34^2*k52*k120+[S]I*F_ES*F_IO^2*F_SK*X^2
*k21*k32*k34^2*k52*k230+[S]I*F_ES*F_IO^3*F_SK*X^3*k21*k32*k34^2*k52*k230+[Na]o*[
R]o*F_NK*F_RK*F_SK*k21*k32^2*k52*k120*k250+[Na]i*F_ES*F_NK*F_RK*F_SK*k21*k3
2*k34*k52^2*k120+[Na]i*F_ES*F_NK*F_RK*F_SK*k21*k32*k34^2*k52*k120+[Na]i*F_ES*F
_NK*F_RK*F_SK*k21*k32^2*k34*k52*k120+[R]o*F_ES*F_NK*F_RK*F_SK*k21*k32*k34^2
*k52*k250+[R]o*F_ES*F_NK*F_RK*F_SK*k21*k32^2*k34*k52*k250+[Na]i*[Na]o*[R]o*F_
NK*F_RK*F_SK*k32^2*k52*k120^2*k250+F_ES*F_IO*F_NK*F_RK*F_SK*X*k21^2*k32*k3
4*k52^2+F_ES*F_IO*F_NK*F_RK*F_SK*X*k21^2*k32*k34^2*k52+F_ES*F_IO*F_NK*F_RK
*F_SK*X*k21^2*k32^2*k34*k52+[Na]o*[R]o*F_NK*F_RK*F_SK*k21*k32*k34*k52*k120*k2
50+[Na]o*[R]o*F_ES*F_IO^2*F_NK*X^2*k21*k32*k34^2*k120*k250+[Na]o*[R]o*F_ES*F_I
O^3*F_NK*X^3*k21*k32*k34^2*k120*k250+[Na]o*[R]o*F_ES*F_IO^2*F_RK*X^2*k32*k34
^2*k52*k120*k250+[Na]o*[S]I*F_ES*F_IO^3*F_SK*X^3*k32*k34^2*k52*k120*k230+[Na]o*
F_ES*F_IO^2*F_NK*F_RK*X^2*k21*k32*k34*k52^2*k120+[Na]o*F_ES*F_IO^2*F_NK*F_R
K*X^2*k21*k32*k34^2*k52*k120+[Na]o*F_ES*F_IO^2*F_NK*F_SK*X^2*k21*k32*k34^2*k5
2*k120+[Na]o*F_ES*F_IO^2*F_NK*F_SK*X^2*k21*k32^2*k34*k52*k120+[R]o*F_ES*F_IO^
2*F_NK*F_RK*X^2*k21*k32*k34^2*k52*k250+[S]I*F_ES*F_IO^2*F_RK*F_SK*X^2*k21*k32

*k34*k52^2*k230+[S]I*F_ES*F_IO^2*F_RK*F_SK*X^2*k21*k32*k34^2*k52*k230+[Na]i*[Na
]o*[R]o*F_NK*F_RK*F_SK*k32*k34*k52*k120^2*k250+[Na]i*[R]o*F_ES*F_NK*F_RK*F_S
K*k32*k34^2*k52*k120*k250+[Na]i*[R]o*F_ES*F_NK*F_RK*F_SK*k32^2*k34*k52*k120*k
250+[Na]i*[S]I*F_ES*F_NK*F_RK*F_SK*k21*k32*k52^2*k120*k230+[Na]i*[S]I*F_ES*F_N
K*F_RK*F_SK*k21*k34*k52^2*k120*k230+[Na]i*[S]I*F_ES*F_NK*F_RK*F_SK*k21*k34^2*
k52*k120*k230+[Na]i*F_ES*F_IO*F_NK*F_RK*X*k21*k32*k34*k52^2*k120+[Na]i*F_ES*F
_IO*F_NK*F_RK*X*k21*k32*k34^2*k52*k120+[Na]i*F_ES*F_IO*F_NK*F_SK*X*k21*k32*k
34^2*k52*k120+[Na]i*F_ES*F_IO*F_NK*F_SK*X*k21*k32^2*k34*k52*k120+[R]o*F_ES*F_I
O*F_NK*F_RK*X*k21*k32*k34^2*k52*k250+[S]I*F_ES*F_IO*F_RK*F_SK*X*k21*k32*k34*
k52^2*k230+[S]I*F_ES*F_IO*F_RK*F_SK*X*k21*k32*k34^2*k52*k230+[Na]o*F_ES*F_IO*F
_NK*F_RK*F_SK*X*k21*k32^2*k52^2*k120+[Na]i*[Na]o*[S]I*F_IO*F_NK*F_RK*F_SK*X*
k32*k52^2*k120^2*k230+[Na]i*[S]I*F_IO^2*F_NK*F_SK*X^2*k21*k32*k34*k52*k120*k230
+[Na]i*[S]I*F_ES*F_NK*F_RK*F_SK*k21*k32*k34*k52*k120*k230+[Na]i*[Na]o*[S]I*F_IO^
2*F_NK*F_SK*X^2*k32*k34*k52*k120^2*k230+[Na]o*[R]o*[S]I*F_ES*F_IO^2*F_SK*X^2*k
32*k34^2*k120*k230*k250+[Na]o*[R]o*[S]I*F_ES*F_IO^3*F_SK*X^3*k32*k34^2*k120*k230
k250+[Na]o[R]o*F_ES*F_IO^2*F_NK*F_SK*X^2*k21*k32*k34^2*k120*k250+[Na]o*[R]o*
F_ES*F_IO^2*F_NK*F_SK*X^2*k21*k32^2*k34*k120*k250+[Na]i*[S]I*F_ES*F_IO^2*F_NK
*F_SK*X^2*k21*k34^2*k52*k120*k230+[Na]i*[S]I*F_ES*F_IO^2*F_NK*F_SK*X^2*k32*k34
^2*k52*k120*k230+[Na]o*[S]I*F_ES*F_IO^2*F_RK*F_SK*X^2*k32*k34*k52^2*k120*k230+[
Na]o*[S]I*F_ES*F_IO^2*F_RK*F_SK*X^2*k32*k34^2*k52*k120*k230+[Na]o*[R]o*F_IO*F_
NK*F_RK*X*k21*k32*k34*k52*k120*k250+[Na]i*[Na]o*[R]o*[S]I*F_NK*F_RK*F_SK*k32*
k52*k120^2*k230*k250+[Na]i*[Na]o*[R]o*[S]I*F_NK*F_RK*F_SK*k34*k52*k120^2*k230*k
250+[Na]i*[R]o*[S]I*F_ES*F_NK*F_RK*F_SK*k34^2*k52*k120*k230*k250+[Na]i*[Na]o*[R]
o*F_IO*F_NK*F_RK*X*k32*k34*k52*k120^2*k250+[Na]i*[R]o*F_ES*F_IO*F_NK*F_RK*X*
k32*k34^2*k52*k120*k250+[Na]o*[R]o*F_ES*F_IO*F_NK*F_SK*X*k21*k32*k34^2*k120*k2
50+[Na]o*[R]o*F_ES*F_IO*F_NK*F_SK*X*k21*k32^2*k34*k120*k250+[Na]o*[R]o*F_ES*F_
IO*F_RK*F_SK*X*k32*k34^2*k52*k120*k250+[Na]o*[R]o*F_ES*F_IO*F_RK*F_SK*X*k32^
2*k34*k52*k120*k250+[Na]i*[S]I*F_ES*F_IO*F_NK*F_SK*X*k21*k34^2*k52*k120*k230+[N
a]i*[S]I*F_IO*F_NK*F_RK*F_SK*X*k21*k32*k52^2*k120*k230+[Na]o*F_ES*F_IO*F_NK*F
_RK*F_SK*X*k21*k32*k34*k52^2*k120+[Na]o*F_ES*F_IO*F_NK*F_RK*F_SK*X*k21*k32*
k34^2*k52*k120+[Na]o*F_ES*F_IO*F_NK*F_RK*F_SK*X*k21*k32^2*k34*k52*k120+[R]o*F
_ES*F_IO*F_NK*F_RK*F_SK*X*k21*k32*k34^2*k52*k250+[R]o*F_ES*F_IO*F_NK*F_RK*
F_SK*X*k21*k32^2*k34*k52*k250+[Na]i*[Na]o*[R]o*[S]I*F_IO*F_NK*F_SK*X*k32*k34*k1
20^2*k230*k250+[Na]i*[Na]o*[S]I*F_IO*F_NK*F_RK*F_SK*X*k32*k34*k52*k120^2*k230+[
Na]i*[R]o*[S]I*F_ES*F_IO*F_NK*F_SK*X*k32*k34^2*k120*k230*k250+[Na]o*[R]o*[S]I*F_
ES*F_IO*F_RK*F_SK*X*k34^2*k52*k120*k230*k250+[Na]o*[R]o*F_ES*F_IO*F_NK*F_RK*
F_SK*X*k21*k32^2*k52*k120*k250+[Na]i*[S]I*F_ES*F_IO*F_NK*F_RK*F_SK*X*k21*k34*
k52^2*k120*k230+[Na]i*[S]I*F_ES*F_IO*F_NK*F_RK*F_SK*X*k21*k34^2*k52*k120*k230+
[Na]i*[S]I*F_ES*F_IO*F_NK*F_RK*F_SK*X*k32*k34*k52^2*k120*k230+[Na]i*[S]I*F_ES*F
_IO*F_NK*F_RK*F_SK*X*k32*k34^2*k52*k120*k230+[Na]o*[R]o*F_ES*F_IO^2*F_NK*F_
RK*X^2*k21*k32*k34*k52*k120*k250+[Na]i*[R]o*[S]I*F_ES*F_NK*F_RK*F_SK*k32*k34*k
52*k120*k230*k250+[Na]i*[Na]o*[R]o*[S]I*F_IO^2*F_NK*F_SK*X^2*k32*k34*k120^2*k23
0*k250+[Na]i*[R]o*[S]I*F_ES*F_IO^2*F_NK*F_SK*X^2*k32*k34^2*k120*k230*k250+[Na]o
[R]o[S]I*F_ES*F_IO^2*F_RK*F_SK*X^2*k34^2*k52*k120*k230*k250+[Na]i*[S]I*F_ES*F_I
O*F_NK*F_SK*X*k21*k32*k34*k52*k120*k230+[Na]i*[S]I*F_IO*F_NK*F_RK*F_SK*X*k21
*k32*k34*k52*k120*k230+[Na]o*[R]o*[S]I*F_ES*F_IO*F_RK*F_SK*X*k32*k34*k52*k120*k
230*k250+[Na]o*[R]o*F_ES*F_IO*F_NK*F_RK*F_SK*X*k21*k32*k34*k52*k120*k250+[Na]
i*[Na]o*[R]o*[S]I*F_IO*F_NK*F_RK*F_SK*X*k32*k52*k120^2*k230*k250+[Na]i*[Na]o*[R]
o*[S]I*F_IO*F_NK*F_RK*F_SK*X*k34*k52*k120^2*k230*k250+[Na]i*[R]o*[S]I*F_ES*F_IO
*F_NK*F_RK*F_SK*X*k34^2*k52*k120*k230*k250+[Na]o*[R]o*[S]I*F_ES*F_IO^2*F_RK*F
_SK*X^2*k32*k34*k52*k120*k230*k250+[Na]i*[R]o*[S]I*F_ES*F_IO*F_NK*F_RK*F_SK*X*
k32*k34*k52*k120*k230*k250))

$\mathbf{B_{mut}-A_{mut}*(1-A_{wt}/B_{wt}) =}$

([S]I*F_IO*F_SK*X*k32*k34*k52*k120*k230*(([Na]i*F_ES*F_NK*k21*(F_IO*k34+F_RK*k34+F_RK*k52))/([R]o*F_RK*k250*([Na]i*[Na]o*F_NK*k120+[Na]o*F_ES*F_IO*k34+[Na]i*F_ES*F_NK*k34))-1)*([Na]i*F_ES*F_NK* F_RK* k21*k34+[Na]i*F_ES*F_NK*F_RK*k21*k52-[Na]i*[Na]o*[R]o*F_NK*F_RK*k120*k250-[Na]i*[R]o*F_ES* F_NK*F_RK*k34*k250+[Na]i*F_ES*F_IO*F_NK*X*k21*k34-[Na]o*[R]o*F_ES*F_IO*F_RK*X*k34*k250))/ (F_ES* F_NK*F_RK*F_SK*k21^2*k32^2*k52^2+F_ES*F_IO^2*F_NK*X^2*k21^2*k32*k34^2*k52+F_ES*F_IO^3*F_NK*X^3*k21^2*k32*k34^2*k52+F_ES*F_NK*F_RK*F_SK*k21^2*k32*k34*k52^2+F_ES*F_NK*F_RK*F_SK*k21^2*k32*k34^2*k52+F_ES*F_NK*F_RK*F_SK*k21^2*k32^2*k34*k52+[Na]i*F_ES*F_NK*F_RK*F_SK*k21*k32^2*k52^2*k120+F_ES*F_IO*F_NK*F_RK*X*k21^2*k32*k34*k52^2+F_ES*F_IO*F_NK*F_RK*X*k21^2*k32*k34^2*k52+F_ES*F_IO*F_NK*F_SK*X*k21^2*k32*k34^2*k52+F_ES*F_IO*F_NK*F_SK*X*k21^2*k32^2*k34*k52+F_ES*F_IO*F_NK*F_RK*F_SK*X*k21^2*k32^2*k52^2+F_ES*F_IO^2*F_NK*F_RK*X^2*k21^2*k32*k34*k52^2+F_ES*F_IO^2*F_NK*F_RK*X^2*k21^2*k32*k34^2*k52+F_ES*F_IO^2*F_NK*F_SK*X^2*k21^2*k32*k34^2*k52+F_ES*F_IO^2*F_NK*F_SK*X^2*k21^2*k32^2*k34*k52+[Na]i*F_ES*F_IO^2*F_NK*X^2*k21*k32*k34^2*k52*k120+[Na]o*F_ES*F_IO^3*F_NK*X^3*k21*k32*k34^2*k52*k120+[S]I*F_ES*F_IO^2*F_SK*X^2*k21*k32*k34^2*k52*k230+[S]I*F_ES*F_IO^3*F_SK*X^3*k21*k32*k34^2*k52*k230+[Na]o*[R]o*F_NK*F_RK*F_SK*k21*k32^2*k52*k120*k250+[Na]i*F_ES*F_NK*F_RK*F_SK*k21*k32*k34*k52^2*k120+[Na]i*F_ES*F_NK*F_RK*F_SK*k21*k32*k34^2*k52*k120+[Na]i*F_ES*F_NK*F_RK*F_SK*k21*k32^2*k34*k52*k120+[R]o*F_ES*F_NK*F_RK*F_SK*k21*k32*k34^2*k52*k250+[R]o*F_ES*F_NK*F_RK*F_SK*k21*k32^2*k34*k52*k250+[Na]i*[Na]o*[R]o*F_NK*F_RK*F_SK*k32^2*k52*k120^2*k250+F_ES*F_IO*F_NK*F_RK*F_SK*X*k21^2*k32*k34*k52^2+F_ES*F_IO*F_NK*F_RK*F_SK*X*k21^2*k32*k34^2*k52+F_ES*F_IO*F_NK*F_RK*F_SK*X*k21^2*k32^2*k34*k52+[Na]o*[R]o*F_NK*F_RK*F_SK*k21*k32*k34*k52*k120*k250+[Na]o*[R]o*F_ES*F_IO^2*F_NK*X^2*k21*k32*k34^2*k120*k250+[Na]o*[R]o*F_ES*F_IO^3*F_NK*X^3*k21*k32*k34^2*k120*k250+[Na]o*[R]o*F_ES*F_IO^2*F_RK*X^2*k32*k34^2*k52*k120*k250+[Na]o*[S]I*F_ES*F_IO^3*F_SK*X^3*k32*k34^2*k52*k120*k230+[Na]o*F_ES*F_IO^2*F_NK*F_RK*X^2*k21*k32*k34*k52^2*k120+[Na]o*F_ES*F_IO^2*F_NK*F_RK*X^2*k21*k32*k34^2*k52*k120+[Na]o*F_ES*F_IO^2*F_NK*F_SK*X^2*k21*k32*k34^2*k52*k120+[Na]o*F_ES*F_IO^2*F_NK*F_SK*X^2*k21*k32^2*k34*k52*k120+[R]o*F_ES*F_IO^2*F_NK*F_RK*X^2*k21*k32*k34^2*k52*k250+[S]I*F_ES*F_IO^2*F_RK*F_SK*X^2*k21*k32*k34*k52^2*k230+[S]I*F_ES*F_IO^2*F_RK*F_SK*X^2*k21*k32*k34^2*k52*k230+[Na]i*[Na]o*[R]o*F_NK*F_RK*F_SK*k32*k34*k52*k120^2*k250+[Na]i*[R]o*F_ES*F_NK*F_RK*F_SK*k32*k34^2*k52*k120*k250+[Na]i*[R]o*F_ES*F_NK*F_RK*F_SK*k32^2*k34*k52*k120*k250+[Na]i*[S]I*F_ES*F_NK*F_RK*F_SK*k21*k32*k52^2*k120*k230+[Na]i*[S]I*F_ES*F_NK*F_RK*F_SK*k21*k34*k52^2*k120*k230+[Na]i*[S]I*F_ES*F_NK*F_RK*F_SK*k21*k34^2*k52*k120*k230+[Na]i*F_ES*F_IO*F_NK*F_RK*X*k21*k32*k34*k52^2*k120+[Na]i*F_ES*F_IO*F_NK*F_RK*X*k21*k32*k34^2*k52*k120+[Na]i*F_ES*F_IO*F_NK*F_SK*X*k21*k32*k34^2*k52*k120+[Na]i*F_ES*F_IO*F_NK*F_SK*X*k21*k32^2*k34*k52*k120+[R]o*F_ES*F_IO*F_NK*F_RK*X*k21*k32*k34^2*k52*k250+[S]I*F_ES*F_IO*F_RK*F_SK*X*k21*k32*k34*k52^2*k230+[S]I*F_ES*F_IO*F_RK*F_SK*X*k21*k32*k34^2*k52*k230+[Na]o*F_ES*F_IO*F_NK*F_RK*F_SK*X*k21*k32^2*k52^2*k120+[Na]i*[Na]o*[S]I*F_IO*F_NK*F_RK*F_SK*X*k32*k52^2*k120^2*k230+[Na]i*[S]I*F_IO^2*F_NK*F_SK*X^2*k21*k32*k34*k52*k120*k230+[Na]i*[S]I*F_ES*F_NK*F_RK*F_SK*k21*k32*k34*k52*k120*k230+[Na]i*[Na]o*[S]I*F_IO^2*F_NK*F_SK*X^2*k32*k34*k52*k120^2*k230+[Na]o*[R]o*[S]I*F_ES*F_IO^2*F_SK*X^2*k32*k34^2*k120*k230*k250+[Na]o*[R]o*[S]I*F_ES*F_IO^3*F_SK*X^3*k32*k34^2*k120*k230*k250+[Na]o*[R]o*F_ES*F_IO^2*F_NK*F_SK*X^2*k21*k32*k34^2*k120*k250+[Na]o*[R]o*F_ES*F_IO^2*F_NK*F_SK*X^2*k21*k32^2*k34*k120*k250+[Na]i*[S]I*F_ES*F_IO^2*F_NK*F_SK*X^2*k21*k34^2*k52*k120*k230+[Na]i*[S]I*F_ES*F_IO^2*F_NK*F_SK*X^2*k32*k34^2*k52*k120*k230+[Na]o*[S]I*F_ES*F_IO^2*F_RK*F_SK*X^2*k32*k34*k52^2*k120*k230+[Na]o*[S]I*F_ES*F_IO^2*F_RK*F_SK*X^2*k32*k34^2*k52*k120*k230+[Na]o*[R]o*F_IO*F_NK*F_RK*X*k21*k32*k34*k52*k120*k250+[Na]i*[Na]o*[R]o*[S]I*F_NK*F_RK*F_SK*k32*k52*k120^2*k230*k250+[Na]i*[Na]o*[R]o*[S]I*F_NK*F_RK*F_SK*k34*k52*k120^2*k230*k250+[Na]i*[R]o*[S]I*F_ES*F_NK*F_R

K*F_SK*k34^2*k52*k120*k230*k250+[Na]i*[Na]o*[R]o*F_IO*F_NK*F_RK*X*k32*k34*k52
*k120^2*k250+[Na]i*[R]o*F_ES*F_IO*F_NK*F_RK*X*k32*k34^2*k52*k120*k250+[Na]o*[R
]o*F_ES*F_IO*F_NK*F_SK*X*k21*k32*k34^2*k120*k250+[Na]o*[R]o*F_ES*F_IO*F_NK*F
_SK*X*k21*k32^2*k34*k120*k250+[Na]o*[R]o*F_ES*F_IO*F_RK*F_SK*X*k32*k34^2*k52*
k120*k250+[Na]o*[R]o*F_ES*F_IO*F_RK*F_SK*X*k32^2*k34*k52*k120*k250+[Na]i*[S]I*F
_ES*F_IO*F_NK*F_SK*X*k21*k34^2*k52*k120*k230+[Na]i*[S]I*F_IO*F_NK*F_RK*F_SK*
X*k21*k32*k52^2*k120*k230+[Na]o*F_ES*F_IO*F_NK*F_RK*F_SK*X*k21*k32*k34*k52^2
*k120+[Na]o*F_ES*F_IO*F_NK*F_RK*F_SK*X*k21*k32*k34^2*k52*k120+[Na]o*F_ES*F_I
O*F_NK*F_RK*F_SK*X*k21*k32^2*k34*k52*k120+[R]o*F_ES*F_IO*F_NK*F_RK*F_SK*X*
k21*k32*k34^2*k52*k250+[R]o*F_ES*F_IO*F_NK*F_RK*F_SK*X*k21*k32^2*k34*k52*k250
+[Na]i*[Na]o*[R]o*[S]I*F_IO*F_NK*F_SK*X*k32*k34*k120^2*k230*k250+[Na]i*[Na]o*[S]I
*F_IO*F_NK*F_RK*F_SK*X*k32*k34*k52*k120^2*k230+[Na]i*[R]o*[S]I*F_ES*F_IO*F_NK
*F_SK*X*k32*k34^2*k120*k230*k250+[Na]o*[R]o*[S]I*F_ES*F_IO*F_RK*F_SK*X*k34^2*k
52*k120*k230*k250+[Na]o*[R]o*F_ES*F_IO*F_NK*F_RK*F_SK*X*k21*k32^2*k52*k120*k2
50+[Na]i*[S]I*F_ES*F_IO*F_NK*F_RK*F_SK*X*k21*k34*k52^2*k120*k230+[Na]i*[S]I*F_E
S*F_IO*F_NK*F_RK*F_SK*X*k21*k34^2*k52*k120*k230+[Na]i*[S]I*F_ES*F_IO*F_NK*F_
RK*F_SK*X*k32*k34*k52^2*k120*k230+[Na]i*[S]I*F_ES*F_IO*F_NK*F_RK*F_SK*X*k32*k
34^2*k52*k120*k230+[Na]o*[R]o*F_ES*F_IO^2*F_NK*F_RK*X^2*k21*k32*k34*k52*k120*
k250+[Na]i*[R]o*[S]I*F_ES*F_NK*F_RK*F_SK*k32*k34*k52*k120*k230*k250+[Na]i*[Na]o
[R]o[S]I*F_IO^2*F_NK*F_SK*X^2*k32*k34*k120^2*k230*k250+[Na]i*[R]o*[S]I*F_ES*F_I
O^2*F_NK*F_SK*X^2*k32*k34^2*k120*k230*k250+[Na]o*[R]o*[S]I*F_ES*F_IO^2*F_RK*F
_SK*X^2*k34^2*k52*k120*k230*k250+[Na]i*[S]I*F_ES*F_IO*F_NK*F_SK*X*k21*k32*k34*
k52*k120*k230+[Na]i*[S]I*F_IO*F_NK*F_RK*F_SK*X*k21*k32*k34*k52*k120*k230+[Na]o
[R]o[S]I*F_ES*F_IO*F_RK*F_SK*X*k32*k34*k52*k120*k230*k250+[Na]o*[R]o*F_ES*F_I
O*F_NK*F_RK*F_SK*X*k21*k32*k34*k52*k120*k250+[Na]i*[Na]o*[R]o*[S]I*F_IO*F_NK*
F_RK*F_SK*X*k32*k52*k120^2*k230*k250+[Na]i*[Na]o*[R]o*[S]I*F_IO*F_NK*F_RK*F_S
K*X*k34*k52*k120^2*k230*k250+[Na]i*[R]o*[S]I*F_ES*F_IO*F_NK*F_RK*F_SK*X*k34^2*
k52*k120*k230*k250+[Na]o*[R]o*[S]I*F_ES*F_IO^2*F_RK*F_SK*X^2*k32*k34*k52*k120*k
230*k250+[Na]i*[R]o*[S]I*F_ES*F_IO*F_NK*F_RK*F_SK*X*k32*k34*k52*k120*k230*k250)
